# Enhancing interpretation of clinical disease-associated copy number variations from multiple sequencing strategies with CNVSeeker

**DOI:** 10.1093/bioinformatics/btag034

**Published:** 2026-01-19

**Authors:** Xudong Xiang, Xinxin Mao, Tengfei Luo, Chenbin Liu, Bozhao Li, Pei Yu, Yu Zhang, Dai Wu, Yijing Wang, Qiao Zhou, Yixiao Zhu, Bin Li, Kun Xia, Guihu Zhao, Jinchen Li

**Affiliations:** Department of Geriatrics, National Clinical Research Centre for Geriatric Disorders, Xiangya Hospital & Centre for Medical Genetics, School of Life Sciences, Central South University, Changsha, Hunan 410008, China; Department of Geriatrics, National Clinical Research Centre for Geriatric Disorders, Xiangya Hospital & Centre for Medical Genetics, School of Life Sciences, Central South University, Changsha, Hunan 410008, China; The Central Laboratory of Birth Defects Prevention and Control & Ningbo Key Laboratory of Genomic Medicine and Birth Defects Prevention, The Affiliated Women and Children’s Hospital, Ningbo University, Ningbo 315000, China; Department of Geriatrics, National Clinical Research Centre for Geriatric Disorders, Xiangya Hospital & Centre for Medical Genetics, School of Life Sciences, Central South University, Changsha, Hunan 410008, China; School of Computer Science and Artificial Intelligence, Hunan University of Technology, Zhuzhou, Hunan 412007, China; Hunan Key Laboratory of Medical Genetics, School of Life Sciences, Central South University, Changsha, Hunan 410008, China; Department of Geriatrics, National Clinical Research Centre for Geriatric Disorders, Xiangya Hospital & Centre for Medical Genetics, School of Life Sciences, Central South University, Changsha, Hunan 410008, China; Hunan Key Laboratory of Medical Genetics, School of Life Sciences, Central South University, Changsha, Hunan 410008, China; Department of Geriatrics, National Clinical Research Centre for Geriatric Disorders, Xiangya Hospital & Centre for Medical Genetics, School of Life Sciences, Central South University, Changsha, Hunan 410008, China; Bioinformatics Center, Xiangya Hospital & Furong Laboratory, Central South University, Changsha, Hunan 410008, China; Department of Geriatrics, National Clinical Research Centre for Geriatric Disorders, Xiangya Hospital & Centre for Medical Genetics, School of Life Sciences, Central South University, Changsha, Hunan 410008, China; Bioinformatics Center, Xiangya Hospital & Furong Laboratory, Central South University, Changsha, Hunan 410008, China; Department of Geriatrics, National Clinical Research Centre for Geriatric Disorders, Xiangya Hospital & Centre for Medical Genetics, School of Life Sciences, Central South University, Changsha, Hunan 410008, China; Bioinformatics Center, Xiangya Hospital & Furong Laboratory, Central South University, Changsha, Hunan 410008, China; Department of Geriatrics, National Clinical Research Centre for Geriatric Disorders, Xiangya Hospital & Centre for Medical Genetics, School of Life Sciences, Central South University, Changsha, Hunan 410008, China; Bioinformatics Center, Xiangya Hospital & Furong Laboratory, Central South University, Changsha, Hunan 410008, China; Department of Neurology, Xiangya Hospital, Central South University, Changsha, Hunan 410008, China; Hunan Key Laboratory of Medical Genetics, School of Life Sciences, Central South University, Changsha, Hunan 410008, China; Department of Geriatrics, National Clinical Research Centre for Geriatric Disorders, Xiangya Hospital & Centre for Medical Genetics, School of Life Sciences, Central South University, Changsha, Hunan 410008, China; Bioinformatics Center, Xiangya Hospital & Furong Laboratory, Central South University, Changsha, Hunan 410008, China; Department of Neurology, Xiangya Hospital, Central South University, Changsha, Hunan 410008, China; Department of Geriatrics, National Clinical Research Centre for Geriatric Disorders, Xiangya Hospital & Centre for Medical Genetics, School of Life Sciences, Central South University, Changsha, Hunan 410008, China; Hunan Key Laboratory of Medical Genetics, School of Life Sciences, Central South University, Changsha, Hunan 410008, China; Bioinformatics Center, Xiangya Hospital & Furong Laboratory, Central South University, Changsha, Hunan 410008, China; Department of Neurology, Xiangya Hospital, Central South University, Changsha, Hunan 410008, China

## Abstract

**Motivation:**

DNA copy number variations (CNVs) exert a profound impact on major genetic disorders in humans. Although multiple sequencing technologies have become the first line of molecular diagnosis for CNVs, existing tools are unable to resolve the pathogenicity of CNVs directly from raw sequencing data.

**Results:**

We developed CNVSeeker, a one-stop and easy-to-use pipeline that provides comprehensive analysis from raw sequencing data to variant interpretation reports, and supports multiple types of sequencing data including short-read data such as whole genome sequencing data and whole exome sequencing data, and long-read sequencing data from Pacific Biosciences HiFi platform or Oxford Nanopore Technologies platform. Through extensive benchmarking, CNVSeeker demonstrated comparable enhancement over the state-of-the-art methods for CNV calling. Moreover, CNVSeeker enables significantly precise variant classification with an accuracy of ∼87%. By applying CNVSeeker to 1946 individuals with autism spectrum disorder (ASD), a total of 133 ASD-associated CNVs in 122 patients were identified, yielding a diagnostic yield of ∼6.3%. Additionally, we have also provided a user-friendly webserver for intuitive visualization of results. This study highlights the potential of CNVSeeker to benefit clinicians and geneticists with limited bioinformatic skill by aiding them interpret CNVs directly from various types of raw sequencing data for auxiliary disease diagnosis.

**Availability and implementation:**

The web server is freely available at https://genemed.tech/cnvseeker and the open-source code can be found at https://github.com/lovelycatZ/CNVSeeker.

## 1 Introduction

Copy number variations (CNVs) represent one of the most prevalent subtypes of structural variations (SVs). They are mainly characterized by unbalanced gain or loss of DNA fragments, ranging in size from over 50 bp to hundreds of mega base pairs (Sudmant *et al.* 2015, Abel *et al.* 2020, Collins *et al.* 2020, Yuan and Jia 2024). In fact, they play a more prominent role than single nucleotide variants (SNVs), accounting for around 4.8%–9.5% of the human genome (Zarrei *et al.* 2015). Consequently, CNVs are often recognized as the significant contributors to various diseases (Weischenfeldt *et al.* 2013), such as cancer (Macintyre *et al.* 2018, Cortés-Ciriano *et al.* 2020), schizophrenia (Marshall *et al.* 2017), developmental delay (Coe *et al.* 2014), autism spectrum disorder (ASD) (Sanders *et al.* 2015), and Parkinson’s disease (Billingsley *et al.* 2023). Thus, further exploration of the pathogenic mechanisms underlying CNVs is imperative for advancing the clinical diagnosis, intervention, and precision medicine of diseases.

The advent of molecular diagnostic techniques, particularly genome sequencing, has revolutionized the field of rare disease diagnosis and health care (Lunke *et al.* 2023). Consequently, numerous analytical methods have been developed, offering diverse capacities such as calling, merging, annotating or interpreting. However, new challenges have hampered their development and application. One key challenge is, different kinds of sequencing technologies such as short-read whole genome sequencing (WGS) and whole exome sequencing (WES), as well as long-read sequencing developed by Pacific Biosciences (PB) or Oxford Nanopore Technologies (ONT), require distinct algorithms for analysis. Existing tools or methods are currently designed and optimized for only one type of sequencing data. Furthermore, the sensitivity of different tools to size, type, and breakpoint resolution of CNVs varies significantly, which makes it challenging to accurately discover genome-wide CNVs through a single method (Kosugi *et al.* 2019). As a result, many studies have adopted multiple algorithms and integrating the output to improve accuracy (Werling *et al.* 2018, Jakubosky *et al.* 2020, Vialle *et al.* 2022, Billingsley *et al.* 2023). And a lot of ensemble callers have also been developed such as MOPlibe, FusorSV, and CN-Learn (Becker *et al.* 2018, Pounraja *et al.* 2019, Kosugi *et al.* 2023). However, the overall accuracy of these tools still requires enhancement. Finally, complete and accurate assessment of pathogenicity of candidate CNVs is sticky. The proposal of American College of Medical Genetics and Genomics (ACMG) and the Clinical Genome Resource (ClinGen) technical standards (Riggs *et al.* 2020) have provided valuable guidance for interpretation and reporting of constitutional CNVs and promoted consistency among different laboratories. However, implementation of the guideline still remains challenge. Firstly, interpretation of large number of CNVs from sequencing data manually is extremely time-consuming and complex. For each scoring criterion, review of different databases and biomedical literature are required to gather relevant evidence, including gene level, variant level and other information. Secondly, the lack of a standardized pipeline can lead to inconsistencies in results across laboratories and clinicians due to the utilization of different tools and databases. Thus, several tools dedicated for interpretation have been developed to tackle these problems such as ClassifyCNV (Gurbich and Ilinsky 2020), AutoCNV (Fan *et al.* 2021), CNV-ClinViewer (Macnee *et al.* 2023), and AnnotSV (Geoffroy *et al.* 2023). They provide either command line tool or web server to facilitate analysis. However, all existing tools are currently limited to analyzing single or multiple CNVs in VCF or BED input format while unable to directly handle raw sequencing data.

**Figure 1:**
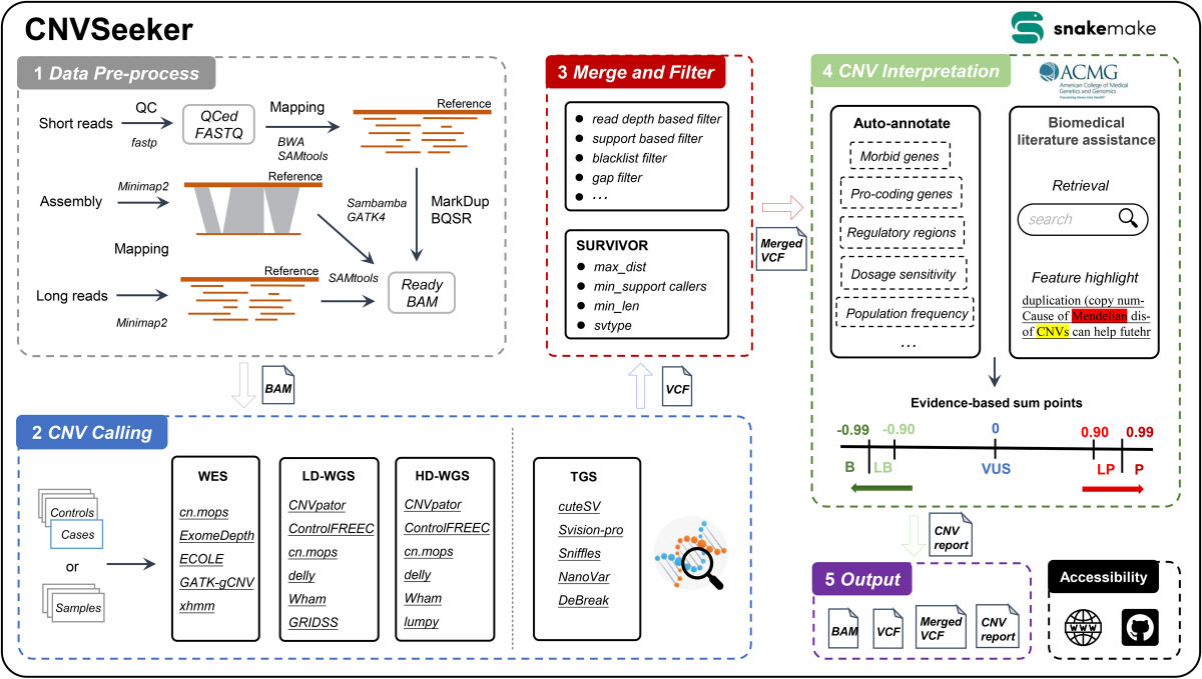
The general framework of CNVSeeker.

In this study, we introduced CNVSeeker (Fig. 1), an ensemble one-stop and easy-to-use pipeline which provides a comprehensive solution for complete analysis of CNVs, spanning from raw sequencing data to variant interpretation report. CNVSeeker offers a wide range of capabilities such as data preprocess, CNV detection, annotation, interpretation, review of biomedical literature and clinical genetics information. By providing robust CNV detection and interpretation, CNVSeeker enables to offer new theoretical support and guidance for genetic counseling, prenatal diagnosis, or other clinical practices.

## 2 Materials and methods

### 2.1 Input description

CNVSeeker accepts a wide range of sequencing data, including short-read whole-genome sequencing data with low- or high-coverage, whole-exome sequencing data and long-read sequencing data generated by Pacific Biosciences and Oxford Nanopore Technologies. Moreover, it supports input data at multiple level. For short-read data, it accepts raw sequencing reads in FASTQ format, alignment reads in SAM/BAM/CRAM format for CNV detection, and CNV call sets in VCF or BED format for interpretation. For long-read data, in addition to formats supported for short reads, CNVSeeker also accepts raw reads in FASTA format, and high-fidelity (HIFI) reads in CCS.BAM format.

CNVSeeker enables to process data based on either GRCh37/hg19 or GRCh38/hg38 human reference genome. As current clinical databases (e.g. gnomAD, DECIPHER) are not yet fully compatible with the T2T reference assembly, CNVSeeker does not provide a complete end-to-end workflow from raw sequencing data to interpretation. Nonetheless, the genomic coordinates are automatically converted to GRCh38/hg38 for downstream analysis if input data are based on T2T reference assembly.

### 2.2 Data preprocessing

A list of analytical tools has been integrated into CNVSeeker to ensure robust data preprocessing (Table 1, available as supplementary data at *Bioinformatics* online). For raw short-read paired-end sequencing data in FASTQ format, quality control and filtering were initially performed using FASTP (Chen 2023). The high-quality reads were then aligned with the human reference genome (build for GRCh37, GRCh38) using the Burrow-Wheeler Aligner (BWA-MEM) (Li and Durbin 2009) to generate read alignments. Afterward, SAMtools was then used to sort the alignments and perform post-alignment filtering. SAMBAMBA (Tarasov *et al.* 2015) was used to mark duplicates sequences introduced by PCR cycles. Finally, the BaseRecalibrator and ApplyBQSR modules of GATK (Babadi *et al.* 2023) was applied to conduct base quality score recalibration (BQSR). Note that this step does not directly improve mapping accuracy, it corrects systematic errors in base quality scores and thereby reducing sequencing noise, which is beneficial to read depth (RD)-based analysis and reads filtering depends on base qualities (Miyatake *et al.* 2015, Talevich *et al.* 2016). For the PacBio circular consensus sequencing (CCS) data, HIFI reads were converted to FASTQ format using the bam2fastq module of the pbtk toolkit. High-quality long reads were aligned with the human reference genome (build for GRCh37, GRCh38) using minimap2 (Li 2018). Default parameters of minimap2 were used, with specific adjustments for sequencing platforms. Specifically, the parameter “-ax” was set to “map-pb” for PacBio continuous long-read datasets, “map-hifi” for PacBio HiFi/CCS datasets, and “map-ont” for Nanopore datasets, respectively. After alignment, BAM files were sorted by genomic coordinates using SAMtools. At this stage, the processed files were ready for downstream analysis.

### 2.3 CNV detection

For both short-read and long-read sequencing data, once the respective preprocessing steps is completed, the resulting BAM files are used as input for the CNV calling module. The selection of specific CNV callers depends on the sequencing platform and coverage level. For high-coverage WGS data, CNVSeeker integrates three read-depth based callers, CNVpytor (Suvakov *et al.* 2021), cn.mops (Klambauer *et al.* 2012), ControlFREEC (Boeva *et al.* 2012), and three read-pair or split-read based caller, delly (Rausch *et al.* 2012), lumpy (Layer *et al.* 2014), and Wham (Kronenberg *et al.* 2015). For low-coverage WGS data, CNVpytor, cn.mops, ControlFREEC, lumpy, delly, Manta (Chen *et al.* 2016), Wham, GRIDSS (Cameron *et al.* 2021) are used. For WES data, the pipeline employs cn.mops, ExomeDepth (Plagnol *et al.* 2012), GATK4, xhmm (Miyatake *et al.* 2015), ECOLE. For long-read sequencing data, cuteSV, SVision-pro, Sniffles (Smolka *et al.* 2024), NanoVar (Tham *et al.* 2020), DeBreak (Chen *et al.* 2023) were used for both PB data and ONT data.

### 2.4 Merging and filtering

After obtaining calls from multiple callers, CNVSeeker firstly performs a series of caller-specific filtering step to remove low confidence signals and reduce artifacts introduced by individual algorithms. For CNVpytor, CNVs with “pytorP1” >0.05, “Q0” >0.9, “pytorRD” >0.75, and “pytorRD” <1.25 were excluded. For ControlFREEC, CNVs with “W” >0.05 or “KS” >0.05 were filtered out. For delly and Manta, CNVs lacking a “PASS” status in either the quality or genotype filter were removed, and CNVs with a genotype of “0/0” were also discarded. For lumpy, CNVs with quality value <120 were removed. For Wham, CNVs with “max_CW” <0.2, or DELs with “TF” <3 for, or DUPs with “U” <2 were filtered out. For Manta, for GRIDSS calls, CNVs annotated with “NO_ASSEMBLY” in the quality filter were removed. For ExomeDepth, CNVs with “BF” <15, or DELs with ratio of observed/expected number of reads >0.8, or DUPs with ratio of observed/expected number of reads <1.1 were filtered out. For GATK4 calls, DELs with copy number state of 0 and 1 were filtered out if quality score fell below min (1000, max(400, 10*nt)) and min (1000, max(100, 10*nt)), respectively. DUPs were removed when their quality score was below min (400, max(50, 4*nt)), where “nt” mean number of exons CNV spans. Notably, to ensure a fair comparison, the same caller-specific filtering criteria were applied in the benchmarking.

Since different CNV callers exhibit variable sensitivities with respect to CNV size ranges, variant types, and breakpoints resolution. CNVSeeker adopts a size- and type-aware merging strategy to achieve optimal performance. We firstly divided CNVs into different types (i.e. DEL and DUP) and size ranges [e.g. SS (50–500 bp), S (500 bp–5 kb), M (5 kb–100 kb), L (>100 kb) for WGS data and long-read sequencing data; S (50 bp–10 kb), M (10 kb–100 kb), L (>100 kb) for WES data]. Within each size range and for each CNV type, we independently evaluated all possible combinations of tools and selected the optimal tool set. By incorporating both size- and type-specific characteristics, CNVSeeker enabled to adopt the most effective strategy for DELs and DUPs across different genomic scales, ultimately achieving superior overall performance (Tables 2 and 3, available as supplementary data at *Bioinformatics* online). Merging calls from multiple CNV callers was performed using SURVIVOR (Jeffares *et al.* 2017) with “max distance between breakpoints” set to 0.5.

Next, blacklist-based filtering and gap-based filtering were performed by CNVSeeker for short-read data: (i) CNVs have 70% reciprocal overlap with region in blacklist were filtered out and (ii) CNVs have 70% reciprocal overlap with region in gap regions were filtered out.

### 2.5 ACMG-based interpretation

CNVSeeker enables users to input the genomic coordinates of a given CNV or upload a bulk of CNVs in VCF or BED format. Before assigning scores, CNVSeeker first need to check whether the query CNV meet the corresponding evidence points by searching and reviewing information from diverse databases.

In this process, a total of nine databases were included for annotation to obtain genomic content, overlap information or gene number of the CNV. Detailed information was shown in Table 4, available as supplementary data at *Bioinformatics* online. A list of 19 260 protein-coding genes was retrieved from HUGO Gene Nomenclature Committee and ENSEMBL to identify genes overlapping with the genomic region of CNV. When multiple members of the same gene family were within a CNV, but none had known disease associations, CNVSeeker considered counting them as single unit to avoid artificially inflating the gene count (Riggs *et al.* 2020). Gene–disease relationships were identified through the Online Mendelian Inheritance in Man (Amberger *et al.* 2015) database. The curated genes and regions from ClinGen were incorporated to establish haploinsufficiency (HI) or benign genes/genomic regions and triplosensitivity or benign genes/genomic regions for DELs and DUPs, respectively. Furthermore, precise coordinates of overlapping genes were reviewed using gene curation from Matched Annotation from NCBI and EMBL-EBI (MANE) project (Morales *et al.* 2022). For those not recorded by MANE, genes with longest transcript in ENSEMBL were considered. The pathogenic variants in the last exon of genes were checked through ClinVar curation. Null variants including nonsense variants, frameshift variants, and splicing variants were determined from ClinVar. For intragenic deletions and duplications, CNVSeeker followed the PVS1 criteria provided by the ClinGen Sequence Variant Interpretation (SVI) working group. We have employed AutoPVS1 (Xiang *et al.* 2020) into CNVSeeker with some enhanced improvement for automated PVS1 annotation. If no established HI gene/genomic region overlaps with the query copy number loss, predicted HI genes were further analyzed with the metric that the deletion involves at least one gene with a gnomAD pLI score ≥0.9 (with the upper bound of the observed/expected confidence interval <0.35) and a DECIPHER HI index (Firth *et al.* 2009) of ≤10%. Lastly, the allele frequency of CNVs in the general population were determined from gnomAD (controls-only dataset, version 2.1), DGV (MacDonald *et al.* 2014), and DECIPHER. All the databases will be automatically updated by CNVSeeker under regular conditions.

## 3 Results

### 3.1 The overview of CNVSeeker and its new features over other methods

Here, we conducted an extensive comparison of CNVSeeker with publicly available tools, pipelines, and web servers dedicated to the analysis, annotation, or evaluation of CNVs (Table 1). Three state-of-the-art ensemble methods, CN-Learn, FusorSV, and MOPline, were primarily limited to CNV detection from raw sequencing data or ready alignment data for a single sequencing technology. It is worth noting that DeAnnCNV (Zhang *et al.* 2015) provided online detection and annotation of CNV from WES datasets, which represents a significant precedent for one-stop analytical workflow. However, it focused solely on processing read counts data and did not support online interpretation. In contrast, CNVSeeker enabled CNV calling from data generated using various sequencing strategies in multiple file format and offered online CNV interpretation based on ACMG/ClinGen guideline. On the other hand, among the popular web servers for CNV interpretation, none were capable of analyzing raw sequencing data. Most of them (such as AutoCNV, AnnotSV, GeneBe) did not support literature review, while REEV and VarSome only provided access to article abstracts. CNVSeeker also exceled in its flexibility and functionality, allowing analysis for batches of CNVs in VCF or BED format, in addition to searching for single or multiple CNV records. It also provided biomedical literatures review associated with queried CNV as well as detailed feature annotation and highlight of full content of interested articles. Lastly, by leveraging Snakemake, tasks involving multiple samples could be processed in parallel, making CNVSeeker highly efficient for large-scale genomic data analysis.

**Table 1:** Comparison of CNVSeeker with other existing tools or pipelines for CNV analysis.

Tool	CNVSeeker	MOPline	FusorSV	CN-Learn	DeAnnCNV	ClassfyCNV	AnnotSV	AutoCNV	GeneBe	REEV	VarSome
Web interface	**✓** ^1^	✗	✗	✗	**✓**	✗	**✓**	**✓**	**✓**	**✓**	**✓**
Commadline interface	**✓**	**✓**	**✓**	**✓**	✗	**✓**	**✓**	**✓**	**✓**	✗	✗
Fastq input	**✓**	✗	**✓**	✗	✗	✗	✗	✗	✗	✗	✗
Data pre-process	**✓**	✗	**✓**	✗	✗	–	–	–	–	–	–
Modify the parameter of ensemble tool through commadline	**✓**	✗	✗	✗	–	–	–	–	–	–	–
Variant calling	**✓**	**✓**	**✓**	**✓**	**✓**	–	–	–	–	–	–
Variant classification (according ACMG guidelines)	**✓**	–	–	**–**	–	**✓**	**✓**	**✓**	**✓**	**✓**	**✓**
Multiple record analysis	**✓**	–	–	–	–	✗	✗	✗	**✓**	✗	✗
File upload analysis	**✓**	–	–	–	–	**✓**	**✓**	**✓**	✗	✗	✗
Multiple file process	**✓**	–	–	–	–	✗	✗	✗	✗	✗	✗
Gene info	**✓**	–	–	–	**✓**	**✓**	**✓**	**✓**	**✓**	**✓**	**✓**
Associated SNVs	**✓**	–	–	–	✗	✗	**✓**	✗	✗	✗	✗
Associated CNVs	**✓**	–	–	–	**✓**	**✓**	**✓**	**✓**	**✓**	**✓**	**✓**
Biomedical literature review	**✓**	–	–	–	✗	✗	✗	✗	✗	**✓** **(abstract only)** ^2^	**✓** **(abstract only)**
Open access	**✓**	**✓**	**✓**	**✓**	**✓**	**✓**	**✓**	**✓**	**✓**	✗	✗
Academic or commercial	Academic	Academic	Academic	Academic	Academic	Academic	Academic	Academic	Academic	Academic	Commercial

1 For visual distinction only.

2 Only the abstract is available.

### 3.2 Benchmarking CNVSeeker with state-of-the-art CNV callers against various types of sequencing data

**Figure 2:**
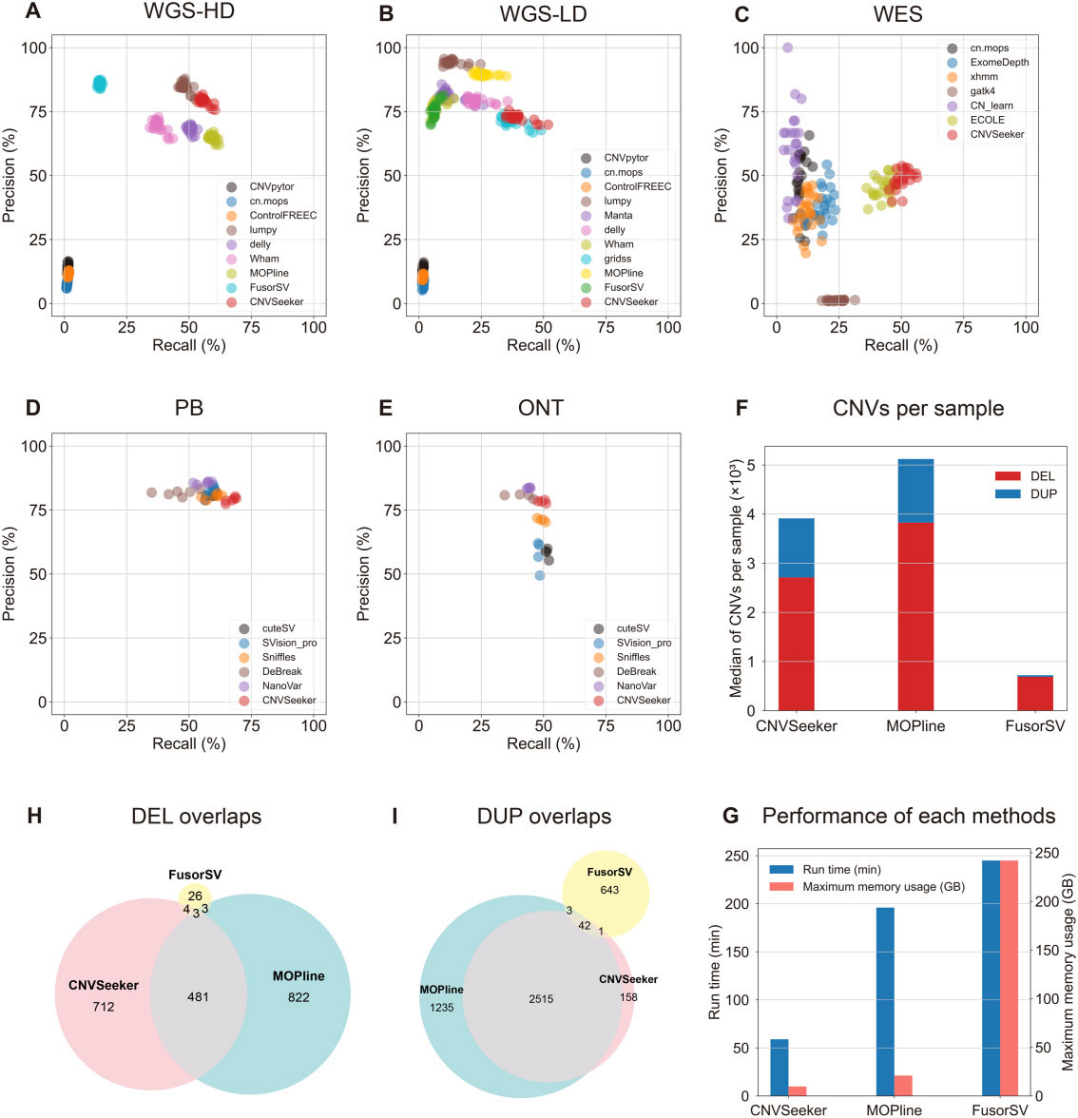
Performance evaluation of CNVSeeker and existing methods using different types of sequencing datasets from 1000 Genome Project (1KGP) and the Human Genome Structural Variation Consortium (HGSVC). CNVs were called by CNVSeeker and other methods on high-coverage WGS data (A), low-coverage WGS data (B), WES data (C), PB data (D), ONT data (E). The precision and recall percentage determined for each CNV type is indicated with the scales on the *x*-axis and *y*-axis, respectively. Each scatter point represents one sample, respectively. (F) CNV calls per sample generated by CNVSeeker and two integrated pipelines, MOPline and FusorSV. CNVs were called using the high-coverage WGS data of 25 samples from 1KGP. (H and I) Overlap of DELs (H) and DUPs (I) among the three pipelines. The numbers in the overlapping regions indicate the counts of shared CNVs. (G) Run time and maximum memory usage of each pipeline evaluated using high-coverage WGS data. All analyses were performed using 24 CPU cores.

We first benchmarked our method against state-of-the-art tools or pipelines using HD-WGS data. CNVSeeker exhibited superior performance for both DEL and DUP categories across almost all CNV size ranges (Fig. 2A; Figs 1A, D and 2, available as supplementary data at *Bioinformatics* online). For deletion calls, CNVSeeker detected more than 60% of true CNVs with up to 90% precision, substantially outperformed the competitors. Although its performance was slightly lower than lumpy within small size range (50–500 bp), CNVSeeker showed greater precision for large fragments (>100 kb) (Fig. 2A and G, available as supplementary data at *Bioinformatics* online). Notably, while FusorSV exhibited marginally higher precision, its recall and the average number calls per sample were significantly lower than those of CNVSeeker, which demonstrate far fewer true positives (Fig. 2A and G). Another ensemble pipeline, MOPline, achieved the highest recall among all methods. However, our method maintained higher precision within most size ranges (Fig. 2A; Fig. 2, available as supplementary data at *Bioinformatics* online). Despite detecting 1094 more DELs and 122 more DUPs than CNVSeeker, MOPline had lower precision, suggesting a greater false-positive rate (Fig. 2A and F). Additionally, both ensemble methods generated a larger proportion of calls not shared with CNVSeeker, likely reflecting their higher number of false-positive or false-negative calls (Fig. 2H and I). Overall, our pipeline enabled to achieve an optimal balance between sensitivity and precision and delivered the highest F-measure among all evaluated methods (Fig. 2A; Table 5, available as supplementary data at *Bioinformatics* online). In terms of runtime, CNVSeeker was significantly faster than both FusorSV and MOPline. Using 24 CPU cores, CNVSeeker required only ∼60 min per sample, compared with FusorSV (∼250 min) and MOPline (∼200 min). The memory usage of CNVSeeker was also significantly lower (Fig. 2G).

**Figure 3:**
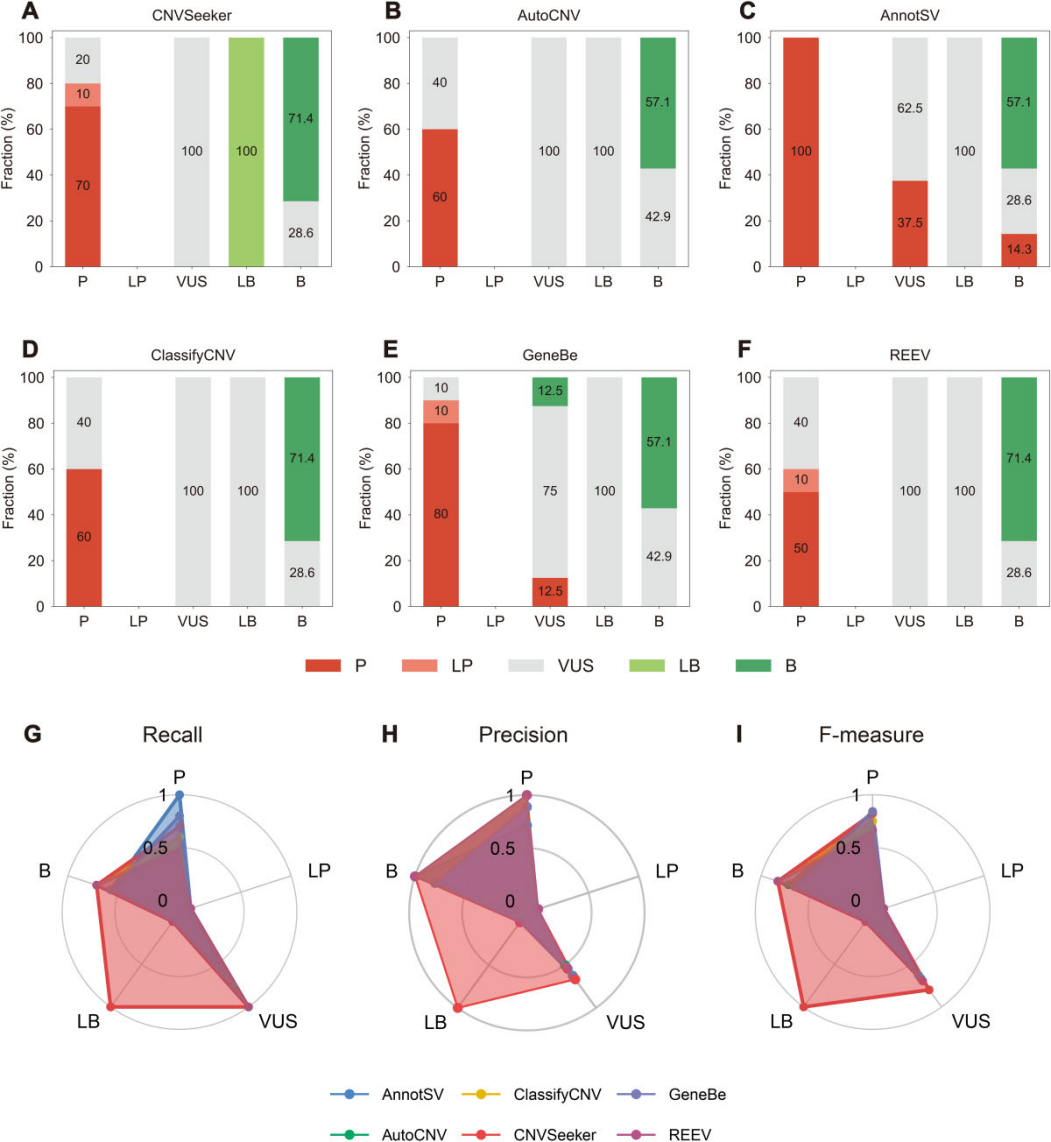
Performance evaluation of CNV classification by different classifiers using 26 illustrative examples from ClinGen. (A–F) Stacked bar plots showing the proportion of CNV classes predicted by each method, including pathogenic (P), likely pathogenic (LP), variant of uncertain significance (VUS), likely benign (LB), and benign (B). Bars represent the fraction of CNVs assigned to each category by CNVSeeker (A), AutoCNV (B), AnnotSV (C), ClassifyCNV (D), GeneBe (E), and REEV (F), respectively. (G–I) Radar charts summarizing the recall (G), precision (H), and F-measure (I) for each tool across the five categories. Colors represent the six methods consistently across all panels.

Consistent with the results from HD-WGS data, CNVSeeker also demonstrated outstanding overall performance using LD-WGS data for both deletions and duplications (Fig. 2B; Fig. 1B and E, available as supplementary data at *Bioinformatics* online). Compared with MOPline, CNVSeeker achieved ∼10% and ∼18% improvement in F-measure values for DEL and DUP, respectively (Table 6, available as supplementary data at *Bioinformatics* online). It also delivered significant enhancement over FusorSV (∼40% for DEL and ∼25% for DUP). Moreover, CNVSeeker enabled to detect a greater number of true-positive CNVs with relatively high precision across nearly all size ranges, particularly for middle and large fragments (Fig. 3, available as supplementary data at *Bioinformatics* online).

**Figure 4:**
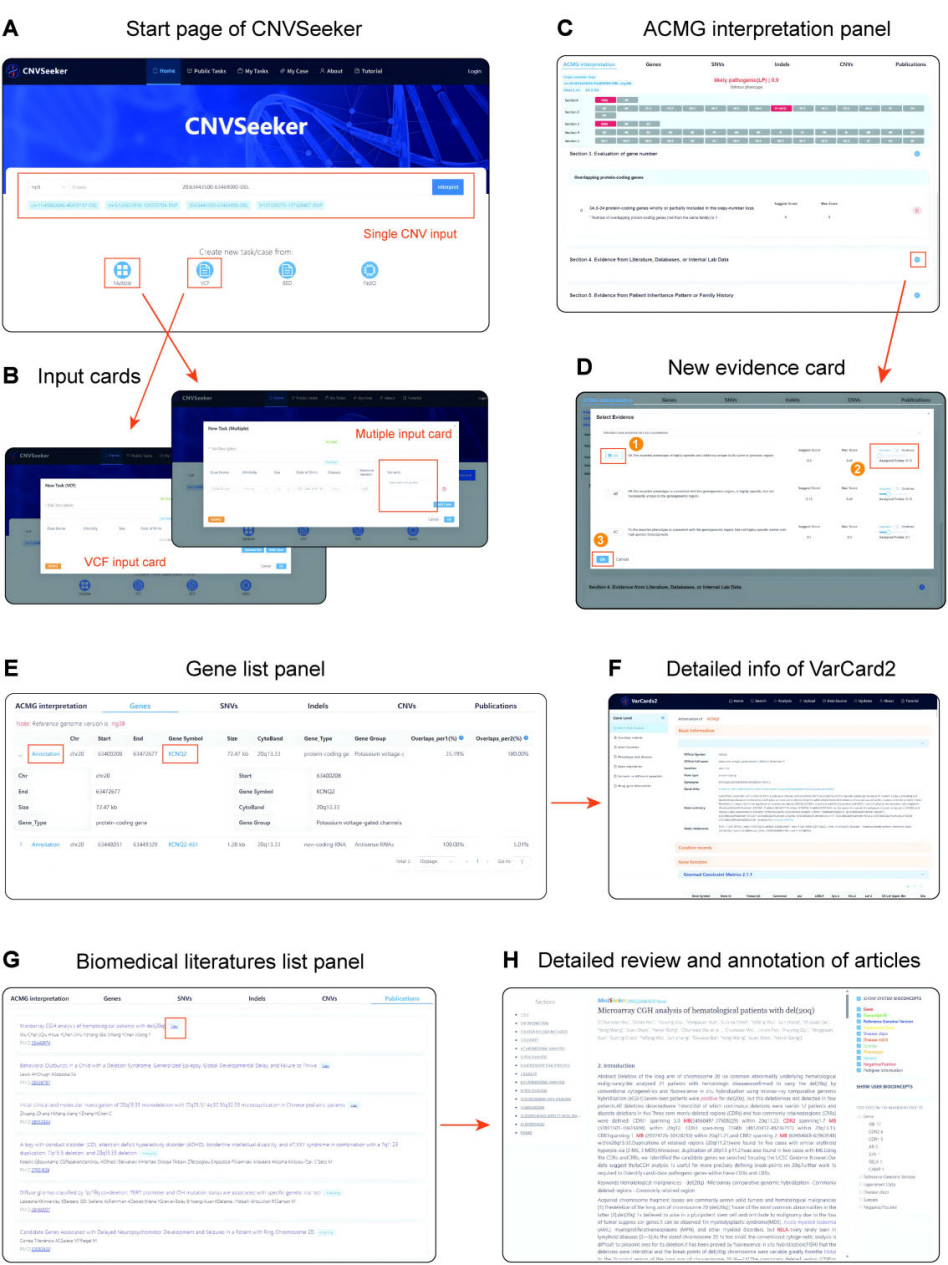
Screenshot of the web interface of CNVSeeker. (A) The start page of CNVSeeker. (B) Input cards of VCF file mode (left) and multiple mode (right). (C) ACMG-based interpretation result page which displays final classification and detailed information. (D) New evidence card for further assessment of CNV depend on users’ own experience. (E) Gene list panel which displays related genes of interested query CNV. (F) Detailed information of gene in the gene list panel provided by VarCards2. (G) Biomedical literature list panel which displays related articles of interested query CNV. (H) Detailed review and feature annotation, highlight of articles provided by the internal tool.

To further enhance practicability, we also integrated an analytical pipeline designed for WES data. When benchmarked against state-of-the-art callers, CNVSeeker achieved the highest overall recall of more than 0.5, indicating higher proportion of true-positive calls (Fig. 2C; Fig. 1C and E, available as supplementary data at *Bioinformatics* online). Compared with the deep learning-based tool ECOLE (Mandiracioglu *et al.* 2024), CNVSeeker showed improved F-measure performance, especially for small and middle DELs. Notably, CNVSeeker achieved exceptional F-measure values for DELs and DUPs that were ∼5 times and ∼6 times higher than another machine learning-based approach CN-learn, respectively (Table 7, available as supplementary data at *Bioinformatics* online). Across different size ranges, CNVSeeker always consistently maintained a balanced recall and precision (Fig. 4, available as supplementary data at *Bioinformatics* online). At the minimum exon-level resolution, CNVSeeker also achieved the best recall and F-measure values, along with robust precision of ∼60% (Fig. 1G–I, available as supplementary data at *Bioinformatics* online).

**Figure 5:**
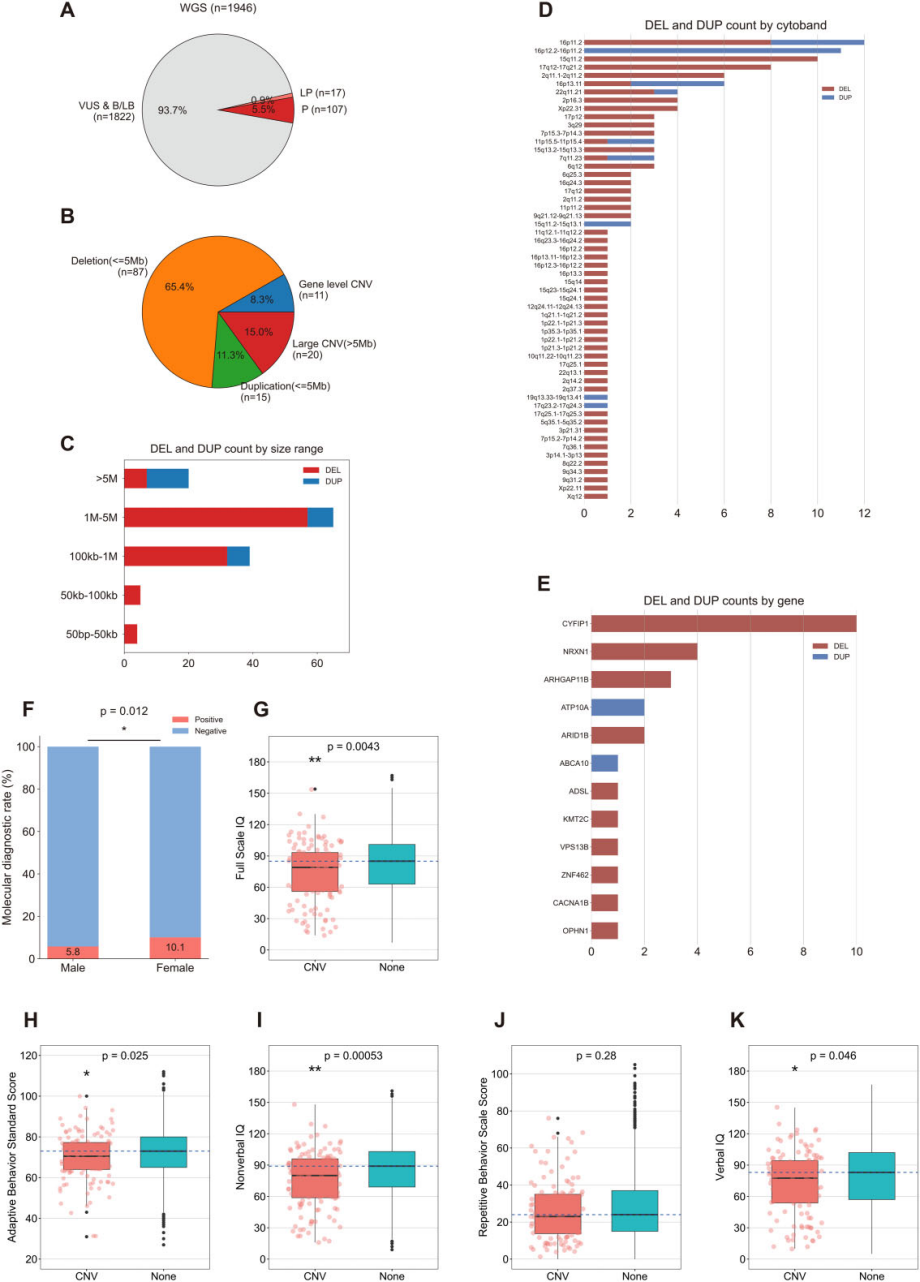
Genomic architecture of CNVs across ASD cohort. (A) Proportion and count of CNV category in the whole ASD cohort with respect to pathogenicity. (B) Proportion and count of CNV category in the whole ASD cohort with respect to size. (C) Distribution of DEL and DUP counts by size range. (D) Distribution of DEL and DUP counts by cytoband location. (E) Distribution of DEL and DUP counts by SFARI gene. (F) Comparison of the diagnostic yield between ASD female and male. (G–K) Distributions of different phenotype measures: (G) full scale IQ, (H) nonverbal IQ, (I) verbal IQ, (J) adaptive behavior standard score, and (K) repetitive behavior scale score for individuals with ASD having ASD-associated CNV, or no CNV. *P < .05; **P≤.01.

CNVSeeker also supports the capacity to analyze long-read sequencing data. On PB data, CNVSeeker achieved the best F-measure of 0.725 and maintained the highest recall across nearly all CNV size ranges while achieving robust precision (Fig. 2D; Fig. 5A, C, E, G and Table 8, available as supplementary data at *Bioinformatics* online). For deletions larger than 100 kb, CNVSeeker provided the highest precision while maintaining recall comparable to other tools. As for ONT data, CNVSeeker likewise achieved the best overall F-measure up to 60.7% and outperformed all other methods in all size ranges (Fig. 2E; Fig. 5B, D, F, H and Table 9, available as supplementary data at *Bioinformatics* online).

### 3.3 CNVSeeker accurately performs interpretation for CNVs

To access the performance of interpretation, we firstly evaluated 26 independent cases from ClinGen illustrative examples using CNVSeeker. As a result, CNVSeeker performed best for consistently classifying 21 out of 26 total CNVs (80.7%) according to expert classifications, including 7 pathogenic (P), 1 likely benign (LB), 5 benign (B), and 8 variants of uncertain significance (VUS), while 18 (69.2%), 19 (73%), 19 (73%), 18 (69.2%), 18 (69.2%) CNVs were classified by AutoCNV, AnnotSV, ClassifyCNV, GeneBe, and REEV, respectively (Fig. 8A–F and Table 10, available as supplementary data at *Bioinformatics* online). In addition, CNVSeeker was the only classifier that succeeded in categorizing LB CNVs correctly (Fig. 3A). Note that two classifiers, AnnotSV and GeneBe, achieved higher consistency compared to CNVSeeker. However, they incorrectly categorized some VUS or B CNVs categories as P category (Fig. 3C and F). Overall, CNVSeeker outperformed other tools in almost all categories with respect to recall, precision, and F-measure (Fig. 3G–I).

Next, we accessed the performance of CNVSeeker on 100 CNVs evaluated by independent reviewers from ACMG/ClinGen original study (Riggs *et al.* 2020). Also, CNVSeeker achieved the highest overall accuracy at 74%, resulting in 11 P, 3 LP, 61 VUS, and 1 B CNVs, while 46 (46%), 69 (69%), 73 (73%) CNVs were classified by AutoCNV, AnnotSV, ClassifyCNV, respectively (Fig. 9A–F and Table 10, available as supplementary data at *Bioinformatics* online). Except for AnnotSV, CNVSeeker performed best for classification of 13 P/LP CNVs (Figs 6A and 9A, available as supplementary data at *Bioinformatics* online). As shown in Fig. 6E–G, available as supplementary data at *Bioinformatics* online, CNVSeeker achieved the best overall recall and F-measure for most categories of CNVs.

We further evaluated the performance of CVNSeeker using 6840 CNVs retrieved from the ClinVar database (accessed on 4 April 2025) consisting of 1545 P, 305 LB, 3 B, 1 LB, and 4986 VUS CNVs, including 3555 deletions and 3285 duplications. As a result, CNVSeeker achieved concordance rates of 71.4% (1103/1545), 7.2% (22/305), 97.7% (4871/4986), and 33.3% (1/3) for the P, LP, VUS, and benign categories, respectively (Figs 7A and 10A, available as supplementary data at *Bioinformatics* online). It could be observed that CNVSeeker significantly outperformed other classifiers in terms of P category except for AnnotSV, which had a higher discordance rate. CNVSeeker also had a lower rate of incorrectly categorizing P as LP, suggesting a more comprehensive classification strategy (Fig. 7A–D, available as supplementary data at *Bioinformatics* online). As shown in Fig. 7E–G, available as supplementary data at *Bioinformatics* online, CNVSeeker featured the best overall recall, precision, and F-measure for most categories of CNVs.

### 3.4 Web-based visualization and user interface of CNVSeeker

In addition to the command-line based end-to-end pipeline, a user-friendly web-based user interface was also provided by CNVSeeker, which was designed to facilitate easy access to analysis and interpretation of CNVs (Fig. 4).

CNVSeeker allowed for simultaneous analysis of single or multiple CNV records/files. For single CNV analysis, it required genomic coordinates in the format “chromosome number: start coordinate-end coordinate-CNV type” (e.g. “chr11:100–200-DEL” or “9:1000–2000-DUP”) based on GRCh37 or GRCh38, with optional related phenotype information (Fig. 4A). As for batch analysis, tasks run in the background, and the status could be checked in the “Public Tasks” page. In multiple mode input card, it required task ID and detailed information of patient. Specifically, a list of CNV records must be provided in the “Variants” field per line. For VCF or BED file mode, the only difference was that the file should be uploaded in the “BED/VCF file” field (Fig. 4B).

After submission, users were subsequently navigated to the result interface which included six different interactive analytical panels. The first panel, labeled “ACMG interpretation,” displayed the results based on ACMG/ClinGen standards (Fig. 4C). By clicking on the rectangle in the table, it would take users to the detailed evidences and scoring metrics. All the evidences were categorized into five different sections, with users able to interactively manage items, modify scores, or remove evidence based on their knowledge or clinical experience. In addition, it also allowed user to add additional evidence items such as the *de novo* status, segregations, or family history that were not automatically implemented by CNVSeeker: (i) click the “+” button, (ii) select the evidence group, (iii) choose the specific evidence item, and (iv) click the “ok” button. This resulted in updated total points and pathogenicity class in the upper part of the first panel (Fig. 4D).

Panels two through five displayed a comprehensive set of genomic, phenotypic, and clinical features of relevant SNVs/Indels, CNVs, and genes. For instance, the second panel detailed the genes associated with the interested CNV (Fig. 4E) providing information including coordinates, gene symbol, size, cytoband location, etc. In addition, by clicking on the “Annotation” or the gene symbol, users were taken to VarCards2 (Wang *et al.* 2024) for in-depth genetic and biomedical information (Fig. 4F).

The last panel displayed publications (Fig. 4G), where CNVSeeker automatically retrieved relevant biomedical literature from PubMed. For open access articles, it employed the internal tool (MedSeeker) to analyze the content and highlight key terms (Fig. 4H). This feature enables geneticists to efficiently gather supplementary evidences and conduct a thorough assessment of CNVs.

### 3.5 Reliable analysis of CNVs from Simons Simplex Collection cohort with CNVSeeker

After extensive method validation for CNV detection and interpretation, we applied CNVSeeker to 1946 individuals obtained from Simons Simplex Collection (SSC) project. We finally identified 133 ASD-associated CNVs in 122 patients. These samples were classified into three main categories including 107 (5.5%) samples with P CNVs, 17 (0.9%) samples with LP CNVs, and 1822 (93.7%) samples with VUS or B/LB CNVs, resulting in overall diagnostic yield of 6.3% (122/1946) (Fig. 5A; Table 11, available as supplementary data at *Bioinformatics* online). The identified CNVs ranged in size from single gene to large fragment abnormalities, which were further subdivided into the following categories: small exonic and gene-level deletions (*n* = 11; 8.3%), deletions ≤5 Mb (*n* = 87; 65.4%), duplications ≤5 Mb (*n* = 15; 11.3%), and large CNV >5 Mb (*n* = 20, 15%) (Fig. 5B). All candidate P/LP CNVs were verified by visualizing using samplot (Belyeu *et al.* 2021).

Regarding the distribution of CNV size ranges, we observed distinct patterns of ASD-associated CNVs, with notable clustering within the range of greater than 5 Mb for both DELs or DUPs. Notably, the count of CNVs in the size range of <100 kb were relatively low (Fig. 5C). To give a detailed view of genomic architecture, we analyzed the impacted genes and regions. The most frequently observed CNVs were located on 16p11.2, 15q11.2, and 17q12-17q21.2 for DELs and 16p11.2, 16p12.2-16p11.2, 16p13.11 for DUPs (Fig. 5D). Among the genes curated in the SFARI project, ASD-associated genes most affected by CNVs included CYFP1, NRXN1, and ARHGAP11B (Fig. 5E). The diagnostic yield of ASD-associated CNVs were 5.8% and 10.1% for males and females, respectively. The results of the statistical test revealed that there was a significant difference between the genders (*P* = .012), indicating that the diagnostic yield of ASD-associated CNVs is higher in female than in male (Fig. 5F).

To explore genotype–phenotype associations, we next compared the distributions of four core consensus phenotype measures of ASD including full scale IQ, nonverbal IQ, verbal IQ, adaptive behavior standard score, and repetitive behavior scale score in individuals with ASD carrying ASD-associated CNVs versus those without such CNVs (Fig. 5G–K). It could be observed that ASD patients with P/LP CNVs were significantly associated with lower scores for phenotypic measures of full scale IQ, adaptive behavior standard score, nonverbal IQ, and verbal IQ. Note that the repetitive behavior scale score was consistently associated.

## 4 Discussion

The ultimate goal of genome sequencing is to accurately evaluate genetic variants, which is the key to understanding the molecular etiology and advancing precision medicine. Despite the growing availability of diverse analytical tools designed for CNV detection, annotation, or interpretation, there are so far no tools that enable complete analysis from sequencing data to variant interpretation reports. We presented CNVSeeker, a one-stop and easy-to-use pipeline tailored for researchers, particularly for clinicians and geneticists with limited bioinformatic skill.

CNVSeeker achieved exceptional overall F-measure for both DEL and DUP categories in almost all CNV size ranges across short-read data and long-read data. Although FusorSV showed the highest overall precision value for high-coverage WGS data, it recovered only a small fraction of true CNVs, resulting in a poor F-measure compared with CNVSeeker. One possible reason for such a low recall value is that FusorSV integrates results from eight tools in its original paper, but we only included seven out of them excluding GenomeSTRiP, for some license issues. Compared to MOPline, CNVSeeker also demonstrated better precision and F-measure value. For WES data, after selecting optimal pairs from different callers, CNVSeeker also achieved best F-measure of 0.495, outperformed the ensemble method CN-Learn and deep learning-based caller ECOLE.

CNVSeeker also achieved excellent performance in variant classification across three different datasets, catching up to 87% overall accuracy on 6840 independent CNVs from ClinVar database. Note that AnnotSV and GeneBe performed better for classifying CNVs of P categories across all datasets. The better performance of these two tools in classifying can be attributed to their incorporation of P/LP CNVs curated from dbVar. AnnotSV determines a CNV as P/LP if the reciprocal overlap exceeded a certain threshold. Although AnnotSV was able to classify P CNVs well, it featured a higher false-positive CNVs by incorrectly classifying B/LB CNV as P/LP, indicating a lack of rigor in its classification method. This lenient condition for reaching P class is problematic because only a small fraction of CNVs in dbVar have been reviewed by experts, with most of the variant statuses being “single submitters” lacking sufficient supporting pathogenic evidence. Note that most of the discordances from ClinVar data occurred in LP or LB/B categories, where they were classified as VUS by CNVSeeker (Fig. 7A, available as supplementary data at *Bioinformatics* online). One possible explanation for these findings is that the CNV classification implemented by CNVSeeker was only based on the results that could be automatically analyzed. Further evaluation based on literature and inheritance pattern was needed to achieve complete classification. However, detailed evidences were not available. Another possible explanation is that some of the classification in ClinVar may be incorrect or incomplete. It has been reported that reevaluation of 246 CNVs in the ClinVar database showed updated clinical classifications in more than 64% of cases (Riggs *et al.* 2018). The discrepancies in assigning CNVs to either pathogenic or benign categories warrant further manual evaluation.

When applied CNVSeeker to 1946 individuals with ASD sourced from quartet families, we identified a total of 133 P or LP ASD-associated CNVs in 122 patients by CNVSeeker, yielding an overall diagnostic yield of 6.3% (122/1946). This rate is higher than that reported in the previous study of the SSC cohort by Trost *et al.* (2022) (5.04%, 122/2419). Finally, genotype-phenotype associations analysis had demonstrated that ASD patient with P/LP CNVs were significantly associated with lower scores for phenotypic measures of full scale IQ and adaptive behavior standard score, which was consistent with previous findings by Trost *et al.* (2022).

In summary, this study introduced a one-stop pipeline to enhance the comprehensive analysis of clinical disease-associated CNVs from multiple sequencing strategies. We believe that it will become benefit to clinicians and geneticists in the auxiliary diagnosis of diseases. CNVSeeker is available in two ways: a commonly used command-line software package that can be run on a local computer or server for offline analysis or a free webserver (https://genemed.tech/cnvseeker/). The source code of CNVSeeker is available on github (https://github.com/lovelycatZ/CNVSeeker).

However, CNVSeeker has several limitations. Firstly, it is designed only for germline CNV analysis from short-read or long-read sequencing but not applicable for cancer research. Secondly, CNVSeeker is still imperfect in its accuracy for detecting CNVs in large repetitive regions with short-read technologies. However, it is a common limitation across all short-read analyses and not exclusive to CNVSeeker.

## Supplementary Material

btag034_Supplementary_Data

## Data Availability

High-coverage raw WGS data (http://ftp-trace.ncbi.nih.gov/1000genomes/ftp/1000G_2504_high_coverage/data) and corresponding SV VCF files (https://ftp.1000genomes.ebi.ac.uk/vol1/ftp/data_collections/1000G_2504_high_coverage/working/20210124.SV_Illumina_Integration/1KGP_3202.Illumina_ensemble_callset.freeze_V1.vcf.gz) were obtained from 1000 genome project. PacBio and ONT raw sequencing data (http://ftp.1000genomes.ebi.ac.uk/vol1/ftp/data_collections/HGSVC3) and corresponding SV VCF (https://ftp.1000genomes.ebi.ac.uk/vol1/ftp/data_collections/HGSVC3/release/Variant_Calls/1.0/GRCh38/) were obtained from Human Genome Structural Variation Consortium. SPARK raw WES datasets and SSC raw WGS datasets can be accessed through SFARIBase (base.sfari.org), accession IDs: SFARI_SSC_WGS_2 and SFARI_DS631850, respectively. Approval by the Simons Foundation for Autism Research Initiative (SFARI) is required. All original code of CNVSeeker has been deposited at (https://github.com/lovelycatZ/CNVSeeker). The detailed protocol of CNVSeeker can be found at (https://github.com/lovelycatZ/CNVSeeker). The resource datasets used for implementation of CNVSeeker can be found at Zenodo (https://doi.org/10.5281/zenodo.15660867).
