# Correction to: Enhancing interpretation of clinical disease-associated copy number variations from multiple sequencing strategies with CNVSeeker

**DOI:** 10.1093/bioinformatics/btag198

**Published:** 2026-05-19

This is a correction to: Xudong Xiang, Xinxin Mao, Tengfei Luo, Chenbin Liu, Bozhao Li, Pei Yu, Yu Zhang, Dai Wu, Yijing Wang, Qiao Zhou, Yixiao Zhu, Bin Li, Kun Xia, Guihu Zhao, Jinchen Li, Enhancing interpretation of clinical disease-associated copy number variations from multiple sequencing strategies with CNVSeeker, Bioinformatics, Volume 42, Issue 2, February 2026, btag034, https://doi.org/10.1093/bioinformatics/btag034

In the originally published version of the paper two co-authors were erroneously additionally marked to be co-corresponding authors on the paper (Bozhao Li and Bin Li). This is now corrected so that co-authors Guihu Zhao and Jinchen Li are the corresponding authors on this paper.

