# Supplementary material for: Enhancing interpretation of clinical disease-associated copy number variations from multiple sequencing strategies with CNVSeeker
Source: Bioinformatics. 2026 Jan 19;42(2):btag034. doi: 10.1093/bioinformatics/btag034 (PMC12918764; doi:10.1093/bioinformatics/btag034)
Supplement: btag034_Supplementary_Data [file btag034_supplementary_data.zip › Supplementary Tables.pdf]

Supplementary Material

for

Enhancing comprehensive analysis of clinical disease-associated copy number variations from multiple sequencing strategies with CNVSeeker

Xiang *et al.*

Table S1. Software used for analyzing or benchmarking.

Software	Version	Source
fastp	v0.2.3	https://github.com/OpenGene/fastp
BWA	v0.7.18	https://github.com/lh3/bwa
samtools	v1.20	https://github.com/samtools/samtools
SAMBAMBA	v1.0.1	https://github.com/biod/sambamba
GATK-gCNV	v4.3.0.0	https://github.com/broadinstitute/gatk
minimap2	v2.28	https://github.com/lh3/minimap2
pbtk	v3.1.1	https://github.com/PacificBiosciences/pbtk
pbmm2	v1.14.99	https://github.com/PacificBiosciences/pbmm2
CNVpytor	v1.3.1	https://github.com/abyzovlab/CNVpytor
cn.mops	v1.48.0	https://www.bioconductor.org/packages/release/bioc/html/cn.mops.html
ControlFREEC	v11.6	https://github.com/BoevaLab/FREEC
delly	v1.2.6	https://github.com/dellytools/delly
Wham	v1.7.0.311	https://github.com/zeeev/wham
smoove	v0.2.8	https://github.com/brentp/smoove
lumpy	v0.3.1	https://github.com/arq5x/lumpy-sv
Manta	v1.6.0	https://github.com/Illumina/manta
GRIDSS2	v2.13.2	https://github.com/PapenfussLab/GRIDSS
ExomeDepth	v1.1.16	https://github.com/vplagnol/ExomeDepth
xhmm	v0.0.0.2016_01_04.cc14e52	https://github.com/RRafiee/XHMM
ECOLE	v0.2	https://github.com/ciceklab/ecole
cuteSV	v2.1.1	https://github.com/tjiangHIT/cuteSV
Sniffles	v2.4	https://github.com/fritzsedlazeck/Sniffles
SVision-pro	v2.4	https://github.com/songbowang125/SVision-pro
NanoVar	v1.7.0	https://github.com/benoukraflab/NanoVar
DeBreak	v1.7.0	https://github.com/Maggi-Chen/DeBreak
AutoPVS1	v2.0	https://github.com/JiguangPeng/autopvs1

FusorSV	git@cd54ffe	https://github.com/timothyjamesbecker/FusorSV
MOPline	v1.8.2	https://github.com/stat-lab/MOPline
CN-Learn	git@2d231cc	https://github.com/girirajanlab/CN_Learn
SURVIVOR	v1.0.7	https://github.com/fritzsedlazeck/SURVIVOR.git
samplot	v1.3.0	https://github.com/ryanlayer/samplot

Table S2. Combination of CNV callers used in CNVSeeker for different sequencing data with respect to CNV size distribution in terms of DELs.

	Combination of tools [1]			
**Data type**	**SS (50bp-500bp)**	**S (500bp-5kb)**	**M (5kb-100kb)**	**L (>100kb)**
WGS-HD	(Wham,delly)(2)+(lumpy)(1)	(delly,lumpy)(2)	(CNVpytor,cn.mops,delly,lumpy)(2)	(CNVpytor,delly,lumpy)(3)+(ControlFREEC,cn.mops)(2)
WGS-LD	(delly,gridss,lumpy)(1)	(delly,gridss,lumpy)(1)	(CNVpytor,cn.mops,delly,lumpy)(2)+(gridss)(1)	(CNVpytor,delly)(2)+(ControlFREEC,cn.mops)(2)
TGS-PB	(DeBreak,Sniffles,cuteSV)(2)+(NanoVar,SVision_pro)(1)	(NanoVar,SVision_pro)(1)	(DeBreak,NanoVar,Sniffles)(2)+(SVision_pro)(1)	(DeBreak,NanoVar,SVision_pro)(2)
TGS-ONT	(DeBreak,NanoVar,cuteSV)(2)+(SVision_pro,Sniffles)(2)	(DeBreak,SVision_pro,Sniffles)(2)+(NanoVar,cuteSV)(2)	(DeBreak,NanoVar,SVision_pro)(2)	(DeBreak,NanoVar)(2)+(SVision_pro,Sniffles,cuteSV)(3)
**Data type**	**-**	**S (50bp-10kb)**	**M (10kb-100kb)**	**L (>100kb)**
WES	-	(ECOLE,ExomeDepth,gatk4,xhmm)(2)+(cn.mops)(1)	(ECOLE,ExomeDepth,cn.mops,gatk4,xhmm)(1)	(ECOLE,gatk4)(1)+(ExomeDepth,xhmm)(1)

[1] (A,B)(m)+(C,D)(n) means: union of calls detected by at least “m” of “A” and “B” plus calls detected by at least “n” of “C” and “D”.

Table S3. Combination of CNV callers used in CNVSeeker for different sequencing data with repect to CNV size distribution in terms of DUPs.

	Combination of tools [1]			
**Data type**	**SS (50bp-500bp)**	**S (500bp-5kb)**	**M (5kb-100kb)**	**L (>100kb)**
WGS-HD	(Wham)(1)+(delly,lumpy)(2)	(Wham,lumpy)(2)+(delly)(1)	(CNVpytor,ControlFREEC)(1)+(Wham,cn.mops,delly,lumpy)(2)	(CNVpytor)(1)+(ControlFREEC,cn.mops)(2)
WGS-LD	(Manta,Wham,gridss)(1)	(Manta,gridss)(1)	(CNVpytor,Manta,Wham)(1)+(ControlFREEC,gridss)(1)	(CNVpytor)(1)+(ControlFREEC,Manta,Wham,cn.mops)(3)
**Data type**	**-**	**S (50bp-10kb)**	**M (10kb-100kb)**	**L (>100kb)**
WES		(ECOLE)(1)+(ExomeDepth)(1)	(ECOLE)(1)+(ExomeDepth,gatk4,xhmm)(2)	(ECOLE,ExomeDepth,cn.mops,xhmm)(1)

[1] (A,B)(m)+(C,D)(n) means: union of calls detected by at least "m" of "A" and "B" plus calls detected by at least "n" of "C" and "D".

Table S4. Databases used for annotation.

**Database**	**Description**	**Source**
HGNC	Gene detail information	https://www.genenames.org
Ensembl	Gene annotation	https://ftp.ensembl.org
NCBI RefSeq MANE Select	Gene annotation	https://ftp.ncbi.nlm.nih.gov/refseq/MANE
ClinGen	HI/TS gene/region curation	https://search.clinicalgenome.org
OMIM	Gene disease association	https:/www.omim.org
ClinVar	Clinical CNV	https://ftp.ncbi.nlm.nih.gov/pub/clinvar
ClinVar	Clinical SNV/InDel	https://ftp.ncbi.nlm.nih.gov/pub/clinvar
gnomAD	SV frequencies	https://gnomad.broadinstitute.org
gnomAD	Gene predicted pLoF metrics	https://gnomad.broadinstitute.org
DECIPHER	Gene HI index	https://www.deciphergenomics.org
DECIPHER	CNV frequencies	https://www.deciphergenomics.org
DGV	Gold standard SV frequencies	https://dgv.tcag.ca/dgv

Table S5. Performance of each method on high depth WGS data with respect to F-measure.

	CNVpytor	cn.mops	ControlFREEC	lumpy	delly	Wham	MOPline	FusorSV	**CNVSeeker**
ALL	0.025	0.019	0.028	0.614	0.581	0.489	0.619	0.243	0.656
DEL	0.014	0.017	0.012	0.737	0.695	0.535	0.728	0.286	0.743
DEL-L	0.061	0.197	0.055	0.045	0.03	0.062	0.075	0.0	0.249
DEL-M	0.093	0.088	0.092	0.564	0.529	0.499	0.586	0.577	0.607
DEL-S	0.0	0.0	0.0	0.733	0.731	0.55	0.737	0.637	0.737
DEL-SS	0.0	0.0	0.0	0.752	0.698	0.527	0.729	0.041	0.749
DUP	0.055	0.024	0.065	0.118	0.182	0.374	0.311	0.007	0.436
DUP-L	0.337	0.093	0.193	0.017	0.021	0.022	0.215	0.226	0.401
DUP-M	0.11	0.054	0.13	0.036	0.061	0.041	0.097	0.043	0.196
DUP-S	0.0	0.0	0.0	0.224	0.294	0.146	0.09	0.0	0.309
DUP-SS	0.0	0.0	0.0	0.097	0.16	0.531	0.457	0.0	0.542

Table S6. Performance of each method on low depth WGS data with respect to F-measure.

	CNVpytor	cn.mops	ControlFREEC	lumpy	Manta	delly	Wham	gridss	MOPline	FusorSV	**CNVSeeker**
ALL	0.029	0.027	0.027	0.231	0.209	0.361	0.124	0.489	0.403	0.118	0.512
DEL	0.017	0.031	0.011	0.301	0.254	0.446	0.133	0.566	0.48	0.14	0.586
DEL-L	0.057	0.136	0.057	0.04	0.097	0.052	0.024	0.035	0.11	0.0	0.172
DEL-M	0.102	0.124	0.071	0.292	0.315	0.44	0.151	0.477	0.441	0.283	0.517
DEL-S	0.0	0.0	0.0	0.356	0.297	0.557	0.173	0.572	0.483	0.368	0.61
DEL-SS	0.0	0.0	0.0	0.278	0.229	0.395	0.115	0.572	0.483	0.017	0.576
DUP	0.062	0.018	0.064	0.008	0.07	0.086	0.1	0.274	0.147	0.012	0.32
DUP-L	0.325	0.003	0.198	0.002	0.037	0.012	0.011	0.012	0.251	0.142	0.368
DUP-M	0.137	0.041	0.137	0.005	0.012	0.042	0.015	0.031	0.042	0.052	0.2
DUP-S	0.0	0.0	0.0	0.019	0.078	0.177	0.042	0.15	0.069	0.0	0.153
DUP-SS	0.0	0.0	0.0	0.004	0.092	0.045	0.165	0.404	0.217	0.0	0.411

Table S7. Performance of each method on WES data with respect to F-measure.

	cn.mops	ExomeDepth	xhmm	gatk4	CN_learn	ECOLE	**CNVSeeker**
ALL	0.161	0.246	0.181	0.023	0.116	0.44	0.495
DEL	0.216	0.315	0.196	0.208	0.142	0.385	0.466
DEL-L	0.026	0.103	0.065	0.024	0.088	0.267	0.317
DEL-M	0.138	0.245	0.142	0.133	0.061	0.146	0.28
DEL-S	0.105	0.1	0.08	0.1	0.0	0.07	0.162
DUP	0.116	0.178	0.167	0.017	0.092	0.481	0.509
DUP-L	0.111	0.11	0.102	0.02	0.125	0.169	0.266
DUP-M	0.057	0.103	0.109	0.035	0.018	0.39	0.408
DUP-S	0.029	0.08	0.043	0.002	0.0	0.102	0.112

Table S8. Performance of each method on PacBio long read data with respect to F-measure in terms of DELs.

	cuteSV	SVision_pro	Sniffles	DeBreak	NanoVar	**CNVSeeker**
DEL	0.682	0.69	0.686	0.584	0.678	0.725
DEL-L	0.093	0.21	0.172	0.225	0.0	0.288
DEL-M	0.651	0.674	0.642	0.658	0.603	0.685
DEL-S	0.643	0.662	0.637	0.571	0.625	0.68
DEL-SS	0.687	0.69	0.692	0.58	0.685	0.729

Table S9. Performance of each method on ONT long read data with respect to F-measure in terms of DELs.

	cuteSV	SVision_pro	Sniffles	DeBreak	NanoVar	**CNVSeeker**
DEL	0.546	0.522	0.582	0.544	0.578	0.607
DEL-L	0.111	0.207	0.31	0.24	0.0	0.252
DEL-M	0.59	0.614	0.591	0.615	0.555	0.618
DEL-S	0.492	0.555	0.553	0.537	0.534	0.603
DEL-SS	0.552	0.509	0.583	0.537	0.583	0.604

Table S10. Performance comparison of CNVSeeker with other tools on three different datasets.

Dataset source	**CNVSeeker** [1]	AnnotSV	AutoCNV	ClassifyCNV	GeneBe	REEV
ClinGen (n=26)	**0.807**	0.730	0.692	0.730	0.692	0.692
ACMG/ClinGen Guideline (n=100)	**0.74**	0.46	0.69	0.73	-	-
ClinVar (n=6840)	**0.876**	0.716	0.81	0.834	-	-

[1] Bolded tools indicate the highest accuracy.

Table S11. ASD-associated CNVs determined by CNVSeeker from 1946 SSC individuals.

Case ID	Sex	CNV ID	Cytoband	Size	Gene count	ACMG/ClinGen classification codes	CNV classification
SSC07951	male	chr1:146994000-148225000-DEL	1q21.1-1q21.2	1.23 Mb	10	1A (0) + 2A (1) + 2G (0) + 3A (0) + 4L (0.15) = 1.15	Pathogenic (P)
SSC12205	male	chr1:28220979-33050963-DEL	1p35.3-1p35.1	4.83 Mb	62	1A (0) + 2H (0.15) + 3C (0.9) + 4L (0.05) = 1.1	Pathogenic (P)
SSC07343	female	chr1:93078000-95853000-DEL	1p22.1-1p21.3	2.78 Mb	17	1A (0) + 2A (1) + 2H (0.15) + 3A (0) = 1.15	Pathogenic (P)
SSC07343	female	chr1:93078800-100609095-DEL	1p22.1-1p21.2	7.53 Mb	34	1A (0) + 2A (1) + 2H (0.15) + 3B (0.45) = 1.6	Pathogenic (P)
SSC07343	female	chr1:98766000-100608000-DEL	1p21.3-1p21.2	1.84 Mb	14	1A (0) + 2A (1) + 3A (0) = 1	Pathogenic (P)
SSC09720	male	chr10:45791355-50132025-DEL	10q11.22-10q11.23	4.34 Mb	40	1A (0) + 2G (0) + 2H (0.15) + 3C (0.9) + 4L (0.15) = 1.2	Pathogenic (P)
SSC12180	male	chr11:44567533-	11p11.2	2.19 Mb	24	1A (0) + 2A (1) + 2H (0.15)	Pathogenic (P)

		46753331-DEL				+ 3A (0) + 4L (0.03) = 1.18	
SSC10984	female	chr11:45927000-47081941-DEL	11p11.2	1.15 Mb	15	1A (0) + 2A (1) + 2H (0.15) + 3A (0) = 1.15	Pathogenic (P)
SSC09359	male	chr11:57069000-60388872-DEL	11q12.1-11q12.2	3.32 Mb	73	1A (0) + 2A (1) + 2H (0.15) + 3C (0.9) = 2.05	Pathogenic (P)
SSC11183	male	chr11:736946-3896137-DEL	11p15.5-11p15.4	3.16 Mb	60	1A (0) + 2A (1) + 2G (0) + 3C (0.9) + 4L (0.05) = 1.95	Pathogenic (P)
SSC09343	male	chr11:737074-3896103-DUP	11p15.5-11p15.4	3.16 Mb	60	1A (0) + 2G (0) + 2L (0) + 3C (0.9) + 4L (0.15) = 1.05	Pathogenic (P)
SSC05872	male	chr11:737115-3896154-DUP	11p15.5-11p15.4	3.16 Mb	60	1A (0) + 2G (0) + 2L (0) + 3C (0.9) + 4L (0.15) = 1.05	Pathogenic (P)
SSC08014	male	chr12:108914686-113697538-DEL	12q24.11-12q24.13	4.78 Mb	63	1A (0) + 2A (1) + 2H (0.15) + 3C (0.9) = 2.05	Pathogenic (P)
SSC07029	female	chr15:22470000-23207000-DEL	15q11.2	737.0 Kb	5	1A (0) + 2A (1) + 3A (0) + 4L (0.15) + 4N (-0.12) = 1.03	Pathogenic (P)
SSC09033	male	chr15:22476000-23052000-DEL	15q11.2	576.0 Kb	4	1A (0) + 2A (1) + 3A (0) + 4L (0.15) + 4N (-0.12) = 1.03	Pathogenic (P)

SSC02782	male	chr15:22572000-23144000-DEL	15q11.2	572.0 Kb	5	1A (0) + 2A (1) + 3A (0) + 4L (0.15) + 4N (-0.12) = 1.03	Pathogenic (P)
SSC05091	male	chr15:22572000-23129000-DEL	15q11.2	557.0 Kb	5	1A (0) + 2A (1) + 3A (0) + 4L (0.15) + 4N (-0.15) = 1	Pathogenic (P)
SSC07461	male	chr15:22572000-23234000-DEL	15q11.2	662.0 Kb	5	1A (0) + 2A (1) + 3A (0) + 4L (0.15) + 4N (-0.1) = 1.05	Pathogenic (P)
SSC05990	male	chr15:22581000-23100000-DEL	15q11.2	519.0 Kb	4	1A (0) + 2A (1) + 3A (0) + 4L (0.15) + 4N (-0.15) = 1	Pathogenic (P)
SSC03788	male	chr15:22590000-23125000-DEL	15q11.2	535.0 Kb	4	1A (0) + 2A (1) + 3A (0) + 4L (0.15) + 4N (-0.15) = 1	Pathogenic (P)
SSC05692	female	chr15:22608000-23126000-DEL	15q11.2	518.0 Kb	4	1A (0) + 2A (1) + 3A (0) + 4L (0.15) + 4N (-0.15) = 1	Pathogenic (P)
SSC04911	female	chr15:22774691-23445651-DEL	15q11.2	670.96 Kb	8	1A (0) + 2A (1) + 3A (0) + 4L (0.15) = 1.15	Pathogenic (P)
SSC06416	male	chr15:22774696-23445637-DEL	15q11.2	670.94 Kb	8	1A (0) + 2A (1) + 3A (0) + 4L (0.15) = 1.15	Pathogenic (P)
SSC05286	male	chr15:22776716-	15q11.2-	5.95 Mb	26	1A (0) + 2A (1) + 2G (0) +	Pathogenic (P)

		28731385-DUP	15q13.1				2L (0) + 3A (0) + 4L (0.15) = 1.15	
SSC12695	female	chr15:24477000-28832000-DUP	15q11.2-15q13.1	4.36 Mb	15		1A (0) + 2A (0.78) + 2L (0) + 3A (0) + 4L (0.15) = 0.93	Likely pathogenic (LP)
SSC07170	male	chr15:30084000-32239000-DEL	15q13.2-15q13.3	2.16 Mb	13		1A (0) + 2A (1) + 2G (0) + 3A (0) + 4L (0.15) = 1.15	Pathogenic (P)
SSC08551	male	chr15:30624000-32329000-DEL	15q13.2-15q13.3	1.71 Mb	7		1A (0) + 2A (1) + 3A (0) + 4L (0.15) = 1.15	Pathogenic (P)
SSC06568	male	chr15:30627000-32594380-DEL	15q13.2-15q13.3	1.97 Mb	10		1A (0) + 2A (1) + 2G (0) + 3A (0) + 4L (0.15) = 1.15	Pathogenic (P)
SSC07048	female	chr15:36258347-37536379-DEL	15q14	1.28 Mb	2		1A (0) + 2A (1) + 3A (0) = 1	Pathogenic (P)
SSC00263	female	chr15:68807392-73556255-DEL	15q23-15q24.1	4.75 Mb	30		1A (0) + 2A (1) + 3B (0.45) + 4L (0.05) = 1.5	Pathogenic (P)
SSC00263	female	chr15:72672000-73861000-DEL	15q24.1	1.19 Mb	9		1A (0) + 2A (1) + 3A (0) + 4L (0.03) = 1.03	Pathogenic (P)

SSC02031	male	chr16:0-1243000-DEL	16p13.3	1.24 Mb	53	1A (0) + 2A (1) + 2G (0) + 3C (0.9) + 4L (0.15) = 2.05	Pathogenic (P)
SSC10776	male	chr16:15030000-16091000-DUP	16p13.11	1.06 Mb	11	1A (0) + 2A (0.86) + 2L (0) + 3A (0) + 4L (0.15) = 1.01	Pathogenic (P)
SSC10932	male	chr16:15030000-16130000-DEL	16p13.11	1.1 Mb	11	1A (0) + 2A (1) + 3A (0) + 4L (0.15) = 1.15	Pathogenic (P)
SSC04857	male	chr16:15180225-18091852-DEL	16p13.11-16p12.3	2.91 Mb	13	1A (0) + 2A (1) + 2G (0) + 3A (0) + 4L (0.15) = 1.15	Pathogenic (P)
SSC02180	male	chr16:15330000-16269000-DUP	16p13.11	939.0 Kb	10	1A (0) + 2A (1) + 2L (0) + 3A (0) + 4L (0.15) = 1.15	Pathogenic (P)
SSC05894	male	chr16:15357000-16339000-DEL	16p13.11	982.0 Kb	11	1A (0) + 2A (1) + 2G (0) + 3A (0) + 4L (0.15) = 1.15	Pathogenic (P)
SSC08168	male	chr16:15366000-16286000-DUP	16p13.11	920.0 Kb	10	1A (0) + 2A (1) + 2L (0) + 3A (0) + 4L (0.15) = 1.15	Pathogenic (P)
SSC12538	male	chr16:15366000-16238000-DUP	16p13.11	872.0 Kb	10	1A (0) + 2A (1) + 2L (0) + 3A (0) + 4L (0.15) = 1.15	Pathogenic (P)
SSC07624	male	chr16:21166752-	16p12.3-	1.26 Mb	16	1A (0) + 2A (1) + 3A (0) + 4L	Pathogenic (P)

		22425480-DEL	16p12.2			(0.03) = 1.03	
SSC02002	male	chr16:21390372-28562672-DUP	16p12.2-16p11.2	7.17 Mb	55	1A (0) + 2G (0) + 2L (0) + 3C (0.9) + 4L (0.15) = 1.05	Pathogenic (P)
SSC09980	male	chr16:21930000-22461000-DEL	16p12.2	531.0 Kb	8	1A (0) + 2A (1) + 3A (0) + 4L (0.15) + 4N (-0.05) = 1.1	Pathogenic (P)
SSC04928	male	chr16:21935384-29107756-DUP	16p12.2-16p11.2	7.17 Mb	65	1A (0) + 2L (0) + 3C (0.9) + 4L (0.15) = 1.05	Pathogenic (P)
SSC02501	male	chr16:21935395-29107750-DUP	16p12.2-16p11.2	7.17 Mb	65	1A (0) + 2L (0) + 3C (0.9) + 4L (0.15) = 1.05	Pathogenic (P)
SSC05091	male	chr16:21935396-29107855-DUP	16p12.2-16p11.2	7.17 Mb	65	1A (0) + 2L (0) + 3C (0.9) + 4L (0.15) = 1.05	Pathogenic (P)
SSC02734	female	chr16:21935397-29107902-DUP	16p12.2-16p11.2	7.17 Mb	65	1A (0) + 2L (0) + 3C (0.9) + 4L (0.15) = 1.05	Pathogenic (P)
SSC09929	male	chr16:21935397-29107698-DUP	16p12.2-16p11.2	7.17 Mb	65	1A (0) + 2L (0) + 3C (0.9) + 4L (0.15) = 1.05	Pathogenic (P)
SSC06434	male	chr16:21935398-29107807-DUP	16p12.2-16p11.2	7.17 Mb	65	1A (0) + 2L (0) + 3C (0.9) + 4L (0.15) = 1.05	Pathogenic (P)

SSC06131	male	chr16:21935399-29107730-DUP	16p12.2-16p11.2	7.17 Mb	65	1A (0) + 2L (0) + 3C (0.9) + 4L (0.15) = 1.05	Pathogenic (P)
SSC00048	male	chr16:21935406-29107828-DUP	16p12.2-16p11.2	7.17 Mb	65	1A (0) + 2L (0) + 3C (0.9) + 4L (0.15) = 1.05	Pathogenic (P)
SSC04071	male	chr16:21935406-29107851-DUP	16p12.2-16p11.2	7.17 Mb	65	1A (0) + 2L (0) + 3C (0.9) + 4L (0.15) = 1.05	Pathogenic (P)
SSC04810	male	chr16:21935406-29107822-DUP	16p12.2-16p11.2	7.17 Mb	65	1A (0) + 2L (0) + 3C (0.9) + 4L (0.15) = 1.05	Pathogenic (P)
SSC07716	male	chr16:28671000-29004000-DEL	16p11.2	333.0 Kb	11	1A (0) + 2A (1) + 2G (0) + 3A (0) + 4L (0.15) = 1.15	Pathogenic (P)
SSC07238	female	chr16:29436000-30230000-DUP	16p11.2	794.0 Kb	35	1A (0) + 2A (1) + 2L (0) + 3A (0) + 4L (0.15) = 1.15	Pathogenic (P)
SSC07624	male	chr16:29436000-30251000-DUP	16p11.2	815.0 Kb	35	1A (0) + 2A (1) + 2L (0) + 3A (0) + 4L (0.15) = 1.15	Pathogenic (P)
SSC08104	female	chr16:29436000-30185000-DUP	16p11.2	749.0 Kb	31	1A (0) + 2A (0.99) + 2L (0) + 3A (0) + 4L (0.15) = 1.14	Pathogenic (P)
SSC05418	male	chr16:29529000-	16p11.2	724.0 Kb	31	1A (0) + 2A (1) + 2L (0) + 3A	Pathogenic (P)

		30253000-DUP				(0) + 4L (0.15) = 1.15	
SSC02001	male	chr16:29538000-30193000-DEL	16p11.2	655.0 Kb	28	1A (0) + 2A (1) + 3B (0.45) + 4L (0.15) = 1.6	Pathogenic (P)
SSC02884	male	chr16:29538000-30196000-DEL	16p11.2	658.0 Kb	29	1A (0) + 2A (1) + 3B (0.45) + 4L (0.15) = 1.6	Pathogenic (P)
SSC04809	male	chr16:29538000-30202000-DEL	16p11.2	664.0 Kb	30	1A (0) + 2A (1) + 3B (0.45) + 4L (0.15) = 1.6	Pathogenic (P)
SSC02982	male	chr16:29541000-30184000-DEL	16p11.2	643.0 Kb	27	1A (0) + 2A (1) + 3B (0.45) + 4L (0.15) = 1.6	Pathogenic (P)
SSC04587	female	chr16:29541000-30189000-DEL	16p11.2	648.0 Kb	27	1A (0) + 2A (1) + 3B (0.45) + 4L (0.15) = 1.6	Pathogenic (P)
SSC05171	male	chr16:29541000-30187000-DEL	16p11.2	646.0 Kb	27	1A (0) + 2A (1) + 3B (0.45) + 4L (0.15) = 1.6	Pathogenic (P)
SSC03574	female	chr16:29559000-30199000-DEL	16p11.2	640.0 Kb	29	1A (0) + 2A (1) + 3B (0.45) + 4L (0.15) = 1.6	Pathogenic (P)
SSC00315	male	chr16:83643000-87808518-DEL	16q23.3-16q24.2	4.17 Mb	38	1A (0) + 2A (1) + 3C (0.9) + 4L (0.08) = 1.98	Pathogenic (P)

SSC02887	male	chr16:89655000-89740084-DEL	16q24.3	85.08 Kb	7	1A (0) + 2D-4 (0.9) + 3A (0) = 0.9	Likely pathogenic (LP)
SS0013009	male	chr16:89769000-89924862-DEL	16q24.3	155.86 Kb	5	1A (0) + 2C-1 (0.9) + 3A (0) + 4L (0.05) = 0.95	Likely pathogenic (LP)
SSC11571	male	chr17:14175000-15572000-DEL	17p12	1.4 Mb	8	1A (0) + 2A (1) + 3A (0) + 4L (0.15) = 1.15	Pathogenic (P)
SSC06501	female	chr17:14187000-15548000-DEL	17p12	1.36 Mb	7	1A (0) + 2A (1) + 3A (0) + 4L (0.15) = 1.15	Pathogenic (P)
SSC08243	female	chr17:14187000-15548000-DEL	17p12	1.36 Mb	7	1A (0) + 2A (1) + 3A (0) + 4L (0.15) = 1.15	Pathogenic (P)
SSC02230	female	chr17:36240000-38077000-DEL	17q12	1.84 Mb	22	1A (0) + 2A (1) + 3A (0) + 4L (0.15) = 1.15	Pathogenic (P)
SSC12853	female	chr17:36243000-38093000-DEL	17q12	1.85 Mb	22	1A (0) + 2A (1) + 3A (0) + 4L (0.15) = 1.15	Pathogenic (P)
SSC04732	male	chr17:37890000-	17q12-	2.39 Mb	55	1A (0) + 2H (0.15) + 3C (0.9)	Pathogenic (P)

		40284531-DEL	17q21.2			= 1.05	
SSC07340	male	chr17:37890000-40246531-DEL	17q12-17q21.2	2.36 Mb	55	1A (0) + 2H (0.15) + 3C (0.9) = 1.05	Pathogenic (P)
SSC07902	male	chr17:37890000-40284544-DEL	17q12-17q21.2	2.39 Mb	55	1A (0) + 2H (0.15) + 3C (0.9) = 1.05	Pathogenic (P)
SSC09904	male	chr17:37890000-40284531-DEL	17q12-17q21.2	2.39 Mb	55	1A (0) + 2H (0.15) + 3C (0.9) = 1.05	Pathogenic (P)
SSC03157	male	chr17:37896000-40288531-DEL	17q12-17q21.2	2.39 Mb	56	1A (0) + 2H (0.15) + 3C (0.9) = 1.05	Pathogenic (P)
SSC07363	female	chr17:37902000-40260531-DEL	17q12-17q21.2	2.36 Mb	55	1A (0) + 2H (0.15) + 3C (0.9) = 1.05	Pathogenic (P)
SSC03421	female	chr17:37914000-40308556-DEL	17q12-17q21.2	2.39 Mb	56	1A (0) + 2H (0.15) + 3C (0.9) = 1.05	Pathogenic (P)
SSC09823	male	chr17:37983000-40343531-DEL	17q12-17q21.2	2.36 Mb	56	1A (0) + 2H (0.15) + 3C (0.9) = 1.05	Pathogenic (P)
SSC09584	male	chr17:60510080-70465049-DUP	17q23.2-17q24.3	9.95 Mb	76	1A (0) + 2A (0.78) + 2L (0) + 3C (0.9) = 1.68	Pathogenic (P)

SSC05089	male	chr17:74239308-78611256-DEL	17q25.1-17q25.3	4.37 Mb	98	1A (0) + 3C (0.9) = 0.9	Likely pathogenic (LP)
SSC10210	male	chr17:75441000-76583951-DEL	17q25.1	1.14 Mb	39	1A (0) + 3C (0.9) = 0.9	Likely pathogenic (LP)
SSC06957	male	chr19:49898279-52433193-DUP	19q13.33-19q13.41	2.53 Mb	83	1A (0) + 2L (0) + 3C (0.9) = 0.9	Likely pathogenic (LP)
SSC09894	male	chr2:120921500-120928088-DEL	2q14.2	6.59 Kb	1	1A (0) + 2E-1 (0.9) + 3A (0) = 0.9	Likely pathogenic (LP)
SSC07687	male	chr2:241653000-241820530-DEL	2q37.3	167.53 Kb	6	1A (0) + 2A (1) + 3A (0) = 1	Pathogenic (P)
SSC11979	male	chr2:50919000-51027845-DEL	2p16.3	108.85 Kb	1	1A (0) + 2E-1 (0.9) + 3A (0) + 4L (0.15) = 1.05	Pathogenic (P)
SSC07045	male	chr2:50928000-	2p16.3	504.39 Kb	1	1A (0) + 2C-1 (0.9) + 3A (0)	Pathogenic (P)

		51432394-DEL				+ 4L (0.15) = 1.05	
SSC08567	male	chr2:51009000-51107144-DEL	2p16.3	98.14 Kb	1	1A (0) + 2E-1 (0.9) + 3A (0) + 4L (0.12) = 1.02	Pathogenic (P)
SSC07880	male	chr2:51015000-51084118-DEL	2p16.3	69.12 Kb	1	1A (0) + 2E-1 (0.9) + 3A (0) + 4L (0.03) = 0.93	Likely pathogenic (LP)
SSC07799	male	chr2:95538595-97042712-DEL	2q11.1-2q11.2	1.5 Mb	23	1A (0) + 2A (1) + 3A (0) + 4L (0.15) = 1.15	Pathogenic (P)
SSC04434	male	chr2:95974325-97572550-DEL	2q11.1-2q11.2	1.6 Mb	25	1A (0) + 2A (1) + 3A (0) + 4L (0.15) = 1.15	Pathogenic (P)
SSC03711	male	chr2:95981477-97579774-DEL	2q11.1-2q11.2	1.6 Mb	25	1A (0) + 2A (1) + 3A (0) + 4L (0.15) = 1.15	Pathogenic (P)
SSC10624	male	chr2:95981477-97579719-DEL	2q11.1-2q11.2	1.6 Mb	25	1A (0) + 2A (1) + 3A (0) + 4L (0.15) = 1.15	Pathogenic (P)
SSC11826	male	chr2:95981477-97579631-DEL	2q11.1-2q11.2	1.6 Mb	25	1A (0) + 2A (1) + 3A (0) + 4L (0.15) = 1.15	Pathogenic (P)
SSC12701	male	chr2:95981480-	2q11.1-	1.6 Mb	25	1A (0) + 2A (1) + 3A (0) + 4L	Pathogenic (P)

		97579675-DEL	2q11.2			(0.15) = 1.15	
SSC04978	female	chr2:96069000-97290571-DEL	2q11.2	1.22 Mb	22	1A (0) + 2A (1) + 3A (0) + 4L (0.15) = 1.15	Pathogenic (P)
SSC10286	male	chr2:96069000-96902000-DEL	2q11.2	833.0 Kb	20	1A (0) + 2A (1) + 3A (0) + 4L (0.15) = 1.15	Pathogenic (P)
SSC04941	male	chr22:18891692-21006773-DEL	22q11.21	2.12 Mb	41	1A (0) + 2A (1) + 2H (0.15) + 3C (0.9) + 4L (0.15) = 2.2	Pathogenic (P)
SSC09044	male	chr22:18897000-20349000-DEL	22q11.21	1.45 Mb	30	1A (0) + 2A (1) + 2H (0.15) + 3B (0.45) + 4L (0.15) = 1.75	Pathogenic (P)
SSC11313	female	chr22:18918000-20964008-DUP	22q11.21	2.05 Mb	37	1A (0) + 2A (1) + 2L (0) + 3B (0.45) + 4L (0.15) = 1.6	Pathogenic (P)
SSC09044	male	chr22:20340000-20686000-DEL	22q11.21	346.0 Kb	4	1A (0) + 2A (1) + 3A (0) = 1	Pathogenic (P)
SSC04305	male	chr22:40101000-40376012-DEL	22q13.1	275.01 Kb	3	1A (0) + 2D-4 (0.9) + 3A (0) = 0.9	Likely pathogenic (LP)

SSC12423	male	chr3:195954000-197562000-DEL	3q29	1.61 Mb	22	1A (0) + 2A (1) + 2H (0.15) + 3A (0) + 4L (0.15) = 1.3	Pathogenic (P)
SSC00902	male	chr3:195993000-197558000-DEL	3q29	1.57 Mb	22	1A (0) + 2A (1) + 2H (0.15) + 3A (0) + 4L (0.15) = 1.3	Pathogenic (P)
SSC07364	male	chr3:195996000-197554000-DEL	3q29	1.56 Mb	22	1A (0) + 2A (1) + 2H (0.15) + 3A (0) + 4L (0.15) = 1.3	Pathogenic (P)
SSC05351	female	chr3:46802705-48210543-DEL	3p21.31	1.41 Mb	15	1A (0) + 2A (1) + 3A (0) = 1	Pathogenic (P)
SSC03827	male	chr3:67083513-72210354-DEL	3p14.1-3p13	5.13 Mb	15	1A (0) + 2A (1) + 3A (0) + 4L (0.08) = 1.08	Pathogenic (P)
SSC04859	male	chr5:171186522-174813083-DEL	5q35.1-5q35.2	3.63 Mb	24	1A (0) + 2A (1) + 2H (0.15) + 3A (0) + 4L (0.03) = 1.18	Pathogenic (P)
SSC12374	male	chr6:156330000-157376000-DEL	6q25.3	1.05 Mb	2	1A (0) + 2A (1) + 3A (0) + 4L (0.08) = 1.08	Pathogenic (P)
SSC09941	male	chr6:157178081-157191761-DEL	6q25.3	13.68 Kb	1	1A (0) + 2E-1 (0.9) + 3A (0) = 0.9	Likely pathogenic (LP)

SSC12392	male	chr6:64812000-67980085-DEL	6q12	3.17 Mb	2	1A (0) + 2C-1 (0.9) + 3A (0) = 0.9	Likely pathogenic (LP)
SSC12506	male	chr6:64956000-65063734-DEL	6q12	107.73 Kb	1	1A (0) + 2E-1 (0.9) + 3A (0) + 4L (0.03) = 0.93	Likely pathogenic (LP)
SSC10210	male	chr6:65235000-65332618-DEL	6q12	97.62 Kb	2	1A (0) + 2E-1 (0.9) + 3A (0) = 0.9	Likely pathogenic (LP)
SSC06266	female	chr7:152307000-152359919-DEL	7q36.1	52.92 Kb	1	1A (0) + 2E-1 (0.9) + 3A (0) = 0.9	Likely pathogenic (LP)
SSC02890	male	chr7:23818618-32959101-DEL	7p15.3-7p14.3	9.14 Mb	56	1A (0) + 2H (0.15) + 3C (0.9) + 4L (0.03) = 1.08	Pathogenic (P)
SSC04278	female	chr7:23818618-32959101-DEL	7p15.3-7p14.3	9.14 Mb	56	1A (0) + 2H (0.15) + 3C (0.9) + 4L (0.03) = 1.08	Pathogenic (P)
SSC06042	male	chr7:23818618-	7p15.3-	9.14 Mb	56	1A (0) + 2H (0.15) + 3C (0.9)	Pathogenic (P)

		32959101-DEL	7p14.3			+ 4L (0.03) = 1.08	
SSC03651	male	chr7:26936126-35660119-DEL	7p15.2-7p14.2	8.72 Mb	52	1A (0) + 2A (1) + 2H (0.15) + 3C (0.9) = 2.05	Pathogenic (P)
SSC06825	male	chr7:73113000-74699000-DUP	7q11.23	1.59 Mb	26	1A (0) + 2A (0.98) + 2L (0) + 3A (0) + 4L (0.15) = 1.13	Pathogenic (P)
SSC02198	male	chr7:73242000-74647463-DUP	7q11.23	1.41 Mb	24	1A (0) + 2A (0.94) + 2L (0) + 3A (0) + 4L (0.15) = 1.09	Pathogenic (P)
SSC11221	male	chr7:74808000-76301000-DEL	7q11.23	1.49 Mb	20	1A (0) + 2A (1) + 2G (0) + 3A (0) + 4L (0.08) = 1.08	Pathogenic (P)
SSC06525	male	chr8:98973000-99177288-DEL	8q22.2	204.29 Kb	1	1A (0) + 2C-1 (0.9) + 3A (0) + 4L (0.03) = 0.93	Likely pathogenic (LP)
SSC02971	male	chr9:106932247-106937738-DEL	9q31.2	5.49 Kb	1	1A (0) + 2E-1 (0.9) + 3A (0) = 0.9	Likely pathogenic (LP)
SSC05301	female	chr9:137787000-138251572-DEL	9q34.3	464.57 Kb	2	1A (0) + 2D-4 (0.9) + 3A (0) + 4L (0.15) = 1.05	Pathogenic (P)

SSC11356	male	chr9:70041000-75759000-DEL	9q21.12-9q21.13	5.72 Mb	19	1A (0) + 2A (1) + 3A (0) + 4L (0.05) = 1.05	Pathogenic (P)
SSC11109	male	chr9:70737000-75219263-DEL	9q21.12-9q21.13	4.48 Mb	16	1A (0) + 2A (1) + 3A (0) + 4L (0.05) = 1.05	Pathogenic (P)
SSC00460	male	chrX:21925804-24277200-DEL	Xp22.11	2.35 Mb	13	1A (0) + 2A (1) + 2H (0.15) + 3A (0) = 1.15	Pathogenic (P)
SSC07902	male	chrX:6677290-7597369-DEL	Xp22.31	920.08 Kb	2	1A (0) + 2C-1 (0.9) + 3A (0) + 4L (0.15) + 4N (-0.15) = 0.9	Likely pathogenic (LP)
SSC12539	male	chrX:6677290-7597369-DEL	Xp22.31	920.08 Kb	2	1A (0) + 2C-1 (0.9) + 3A (0) + 4L (0.15) + 4N (-0.15) = 0.9	Likely pathogenic (LP)
SSC05030	female	chrX:6741211-8137891-DEL	Xp22.31	1.4 Mb	4	1A (0) + 2A (1) + 3A (0) + 4L (0.15) + 4N (-0.15) = 1	Pathogenic (P)
SSC09894	male	chrX:6741211-8137891-DEL	Xp22.31	1.4 Mb	4	1A (0) + 2A (1) + 3A (0) + 4L (0.15) + 4N (-0.15) = 1	Pathogenic (P)
SSC02602	male	chrX:68190000-	Xq12	34.56 Kb	1	1A (0) + 2E-1 (0.9) + 3A (0)	Likely

68224564-DEL		= 0.9	pathogenic<br>(LP)
