# Supplementary material for: Enhancing interpretation of clinical disease-associated copy number variations from multiple sequencing strategies with CNVSeeker
Source: Bioinformatics. 2026 Jan 19;42(2):btag034. doi: 10.1093/bioinformatics/btag034 (PMC12918764; doi:10.1093/bioinformatics/btag034)
Supplement: btag034_Supplementary_Data [file btag034_supplementary_data.zip › Supplementary Figures.pdf]

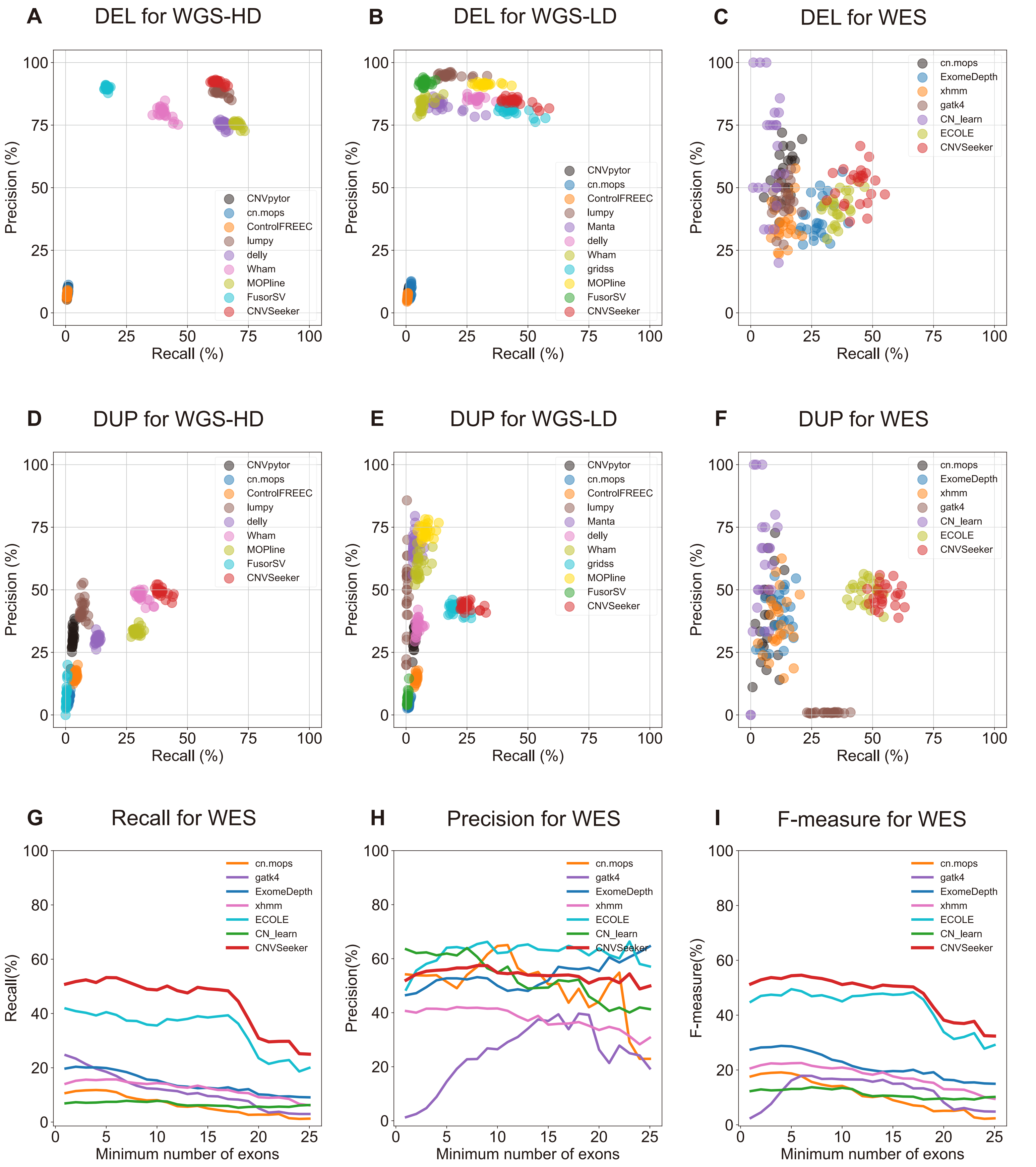

A
DEL for WGS-HD
B
DEL for WGS-LD
C
DEL for WES
D
DUP for WGS-HD
E
DUP for WGS-LD
F
DUP for WES
G
Recall for WES
H
Precision for WES
I
F-measure for WES
Precision (%)
Recall (%)
Recall(%)
Precision(%)
F-measure(%)
Minimum number of exons
CNVpytor
cn.mops
ControlFREEC
lumpy
Manta
delly
Wham
gridss
MOPline
FusorSV
CNVSeeker
ExomeDepth
xhmm
gatk4
CN_learn
ECOLE

Figure S1. Performance evaluation of CNVSeeker and existing methods using different types of sequencing datasets. DEL (**A, B, C**) and DUP (**D, E, F**) were called by CNVSeeker and other methods on high coverange WGS data (**A, D**), low coverage WGS data (**B, E**) and WES data (**C, F**). The precision and recall percentage determined for each CNV type is indicated with the scales on the x-axis and y-axis, respectively. Each scatter point represents one sample, respectively.

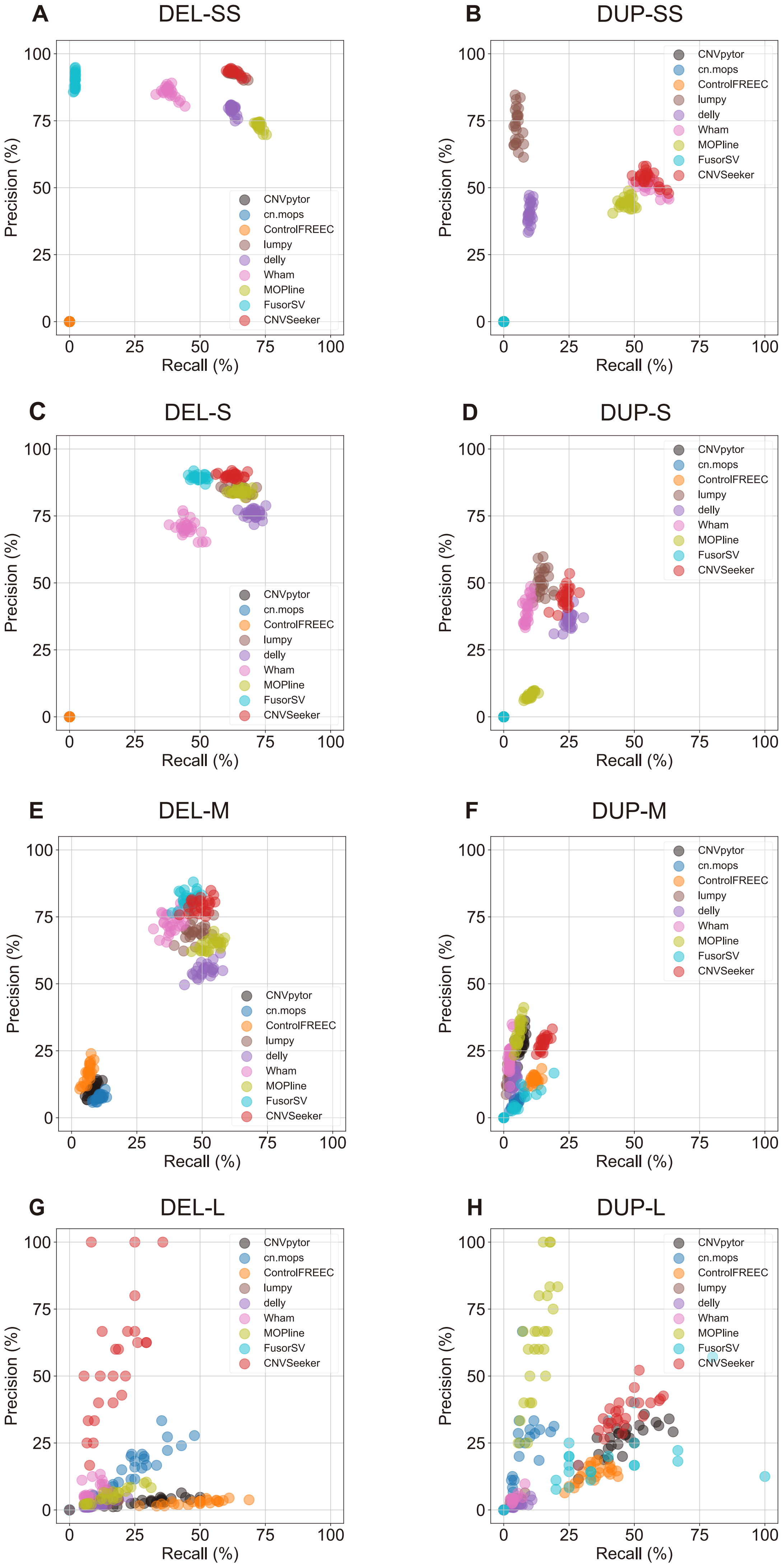

A
DEL-SS
B
DUP-SS
C
DEL-S
D
DUP-S
E
DEL-M
F
DUP-M
G
DEL-L
H
DUP-L
Precision (%)
Recall (%)
0
25
50
75
100
CNVpytor
cn.mops
ControlFREEC
lumpy
delly
Wham
MOPline
FusorSV
CNVSeeker

Figure S2. Performance evaluation of CNVSeeker and existing methods using high coverage WGS datasets in terms of size-stratified DELs and DUPs. DELs (**A, C, E, G**) and DUPs (**B, D, F, H**) were called by CNVSeeker and other methods and were categorized into four size ranges, SS [50 bp−500 bp] (**A, B**), S [500 bp - 5 kb] (**C, D**), M [5 kb −100 kb] (**E, F**), L [>100 kb] (**G, H**). The precision and recall percentages determined for the respective CNV size range are indicated with the scales on the x-axis and y-axis, respectively. Each scatter point represents one sample, respectively.

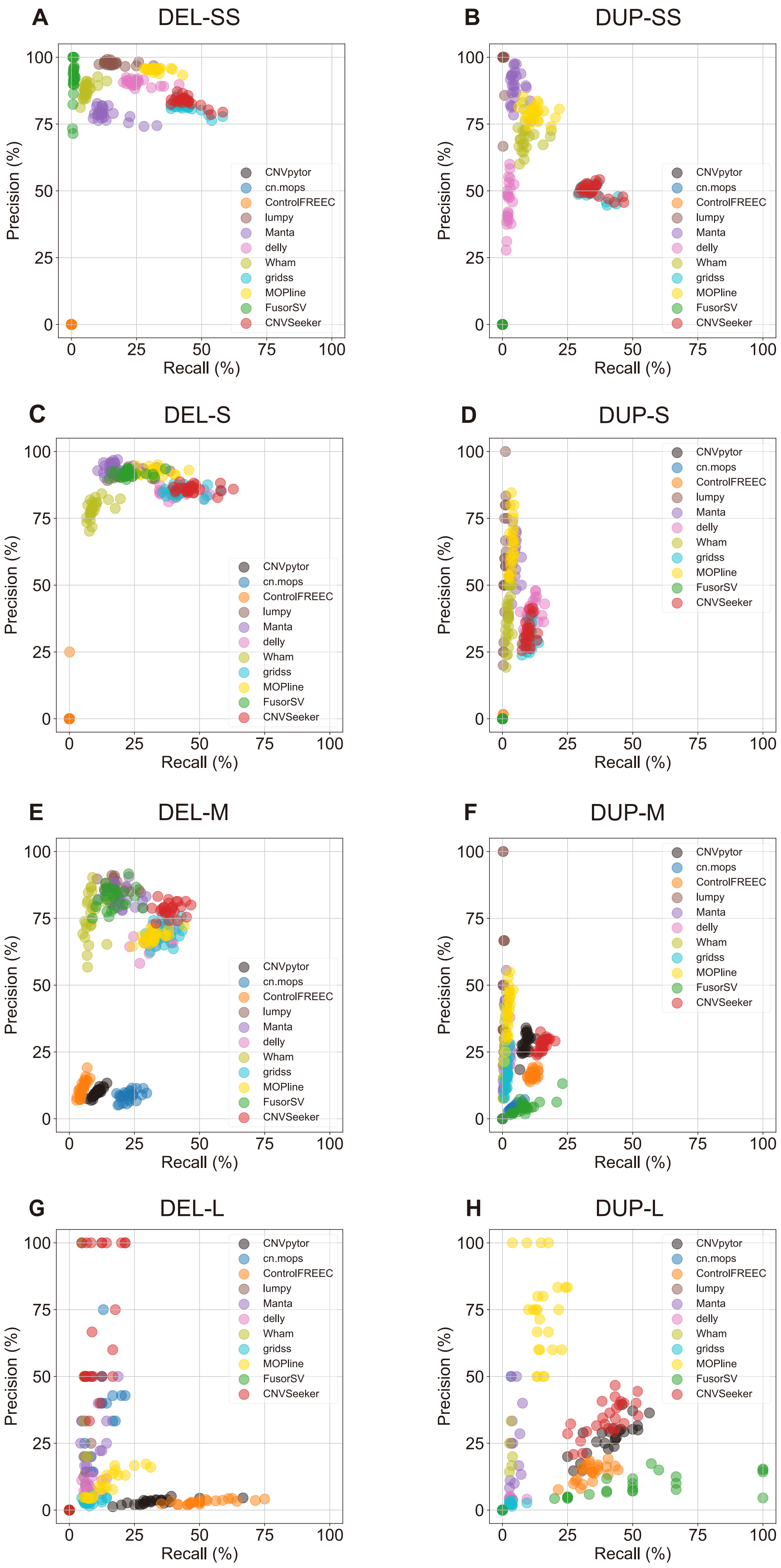
A
DEL-SS
B
DUP-SS
C
DEL-S
D
DUP-S
E
DEL-M
F
DUP-M
G
DEL-L
H
DUP-L
Precision (%)
Recall (%)
CNVpytor
cn.mops
ControlFREEC
lumpy
Manta
delly
Wham
gridss
MOPline
FusorSV
CNVSeeker

Figure S3. Performance evaluation of CNVSeeker and existing methods using low coverage WGS datasets in terms of size-stratified DELs and DUPs. DELs (**A, C, E, G**) and DUPs (**B, D, F, H**) were called by CNVSeeker and other methods and were categorized into four size ranges, SS [50 bp−500 bp] (**A, B**), S [500 bp - 5 kb] (**C, D**), M [5 kb −100 kb] (**E, F**), L [>100 kb] (**G, H**). The precision and recall percentages determined for the respective CNV size range are indicated with the scales on the x-axis and y-axis, respectively. Each scatter point represents one sample, respectively.

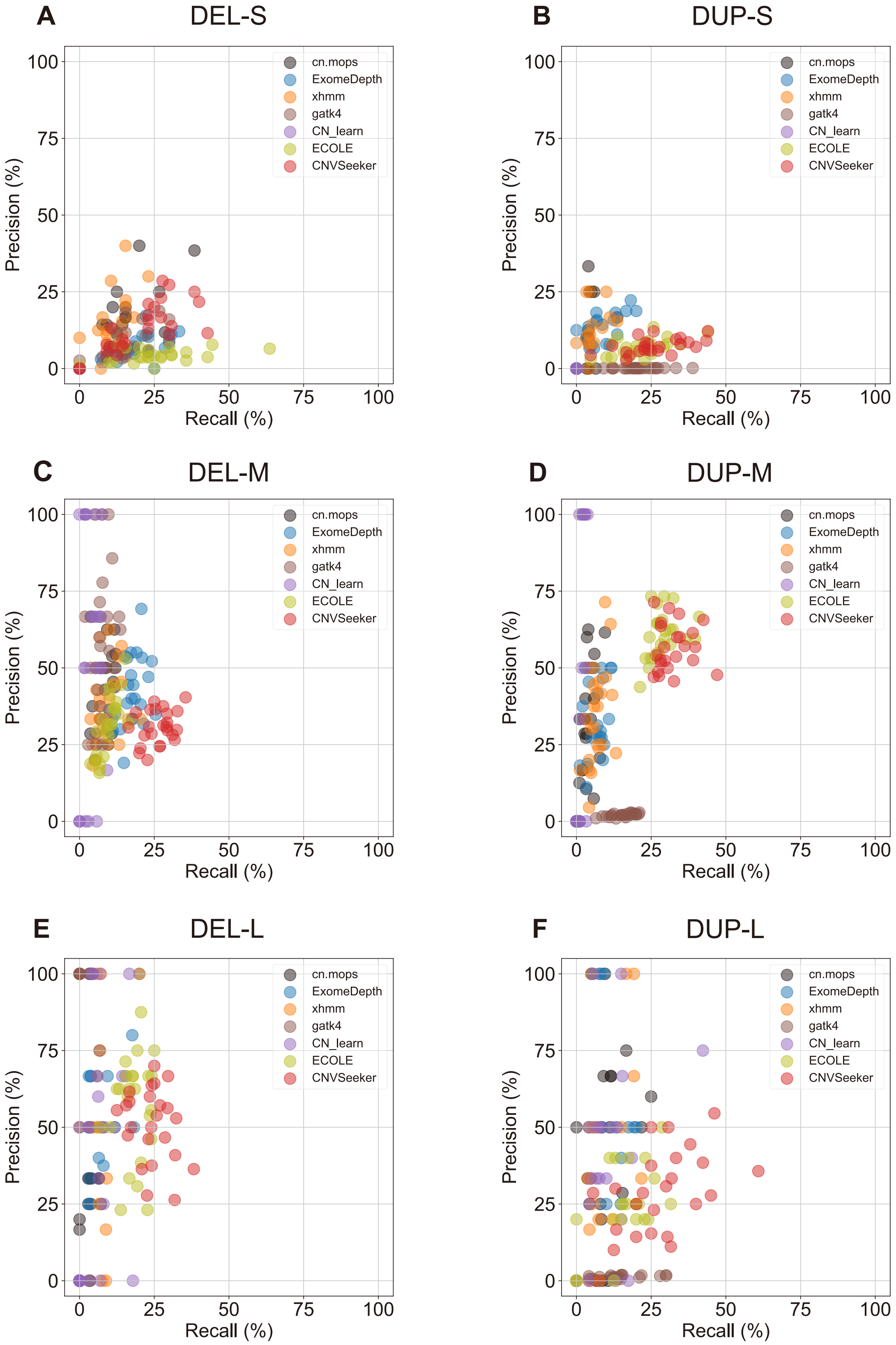
A
DEL-S
B
DUP-S
C
DEL-M
D
DUP-M
E
DEL-L
F
DUP-L
Precision (%)
Recall (%)
0
25
50
75
100
cn.mops
ExomeDepth
xhmm
gatk4
CN_learn
ECOLE
CNVSeeker

Figure S4. Performance evaluation of CNVSeeker and existing methods using WES datasets in terms of size-stratified DELs and DUPs. DELs (**A, C, E**) and DUPs (**B, D, F**) were called by CNVSeeker and other methods and were categorized into three size ranges, S [50 bp −10 kb] (**A, B**), M [10 kb −100 kb] (**C, D**), L [>100 kb] (**E, F**). The precision and recall percentages determined for the respective CNV size range are indicated with the scales on the x-axis and y-axis, respectively. Each scatter point represents one sample, respectively.

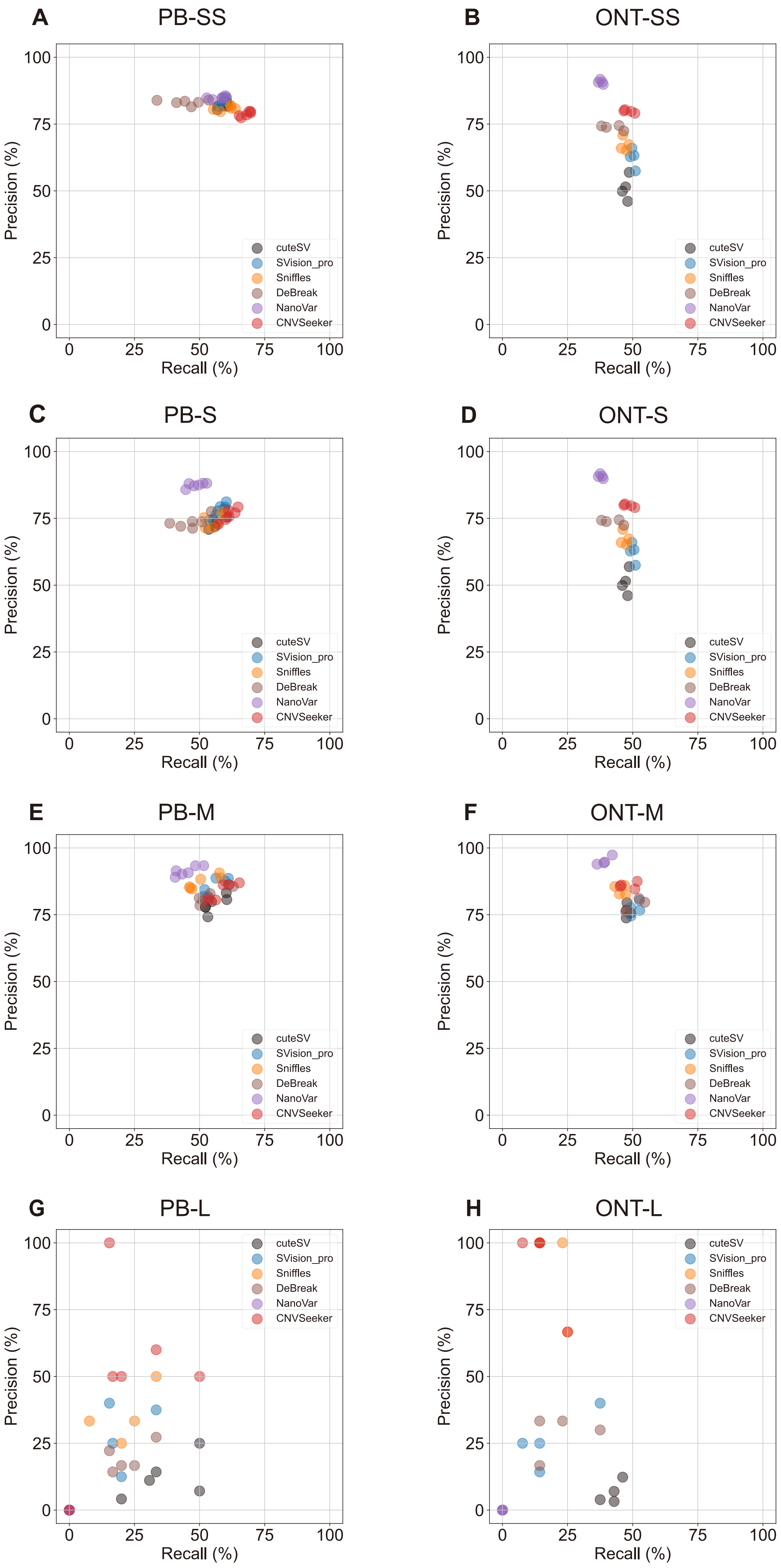
A
PB-SS
B
ONT-SS
C
PB-S
D
ONT-S
E
PB-M
F
ONT-M
G
PB-L
H
ONT-L
Precision (%)
Recall (%)
0
25
50
75
100
cuteSV
SVision_pro
Sniffles
DeBreak
NanoVar
CNVSeeker

Figure S5. Performance evaluation of CNVSeeker and existing methods using long-read sequencing datasets in terms of size-stratified DELs. DELs were called by CNVSeeker and other methods on PB (**A, C, E, G**) and ONT (**B, D, F, H**) datasets and were categorized into four size ranges, SS [50 bp−500 bp] (**A, B**), S [500 bp - 5 kb] (**C, D**), M [5 kb −100 kb] (**E, F**), L [>100 kb] (**G, H**). The precision and recall percentages determined for the respective CNV size range are indicated with the scales on the x-axis and y-axis, respectively. Each scatter point represents one sample, respectively.

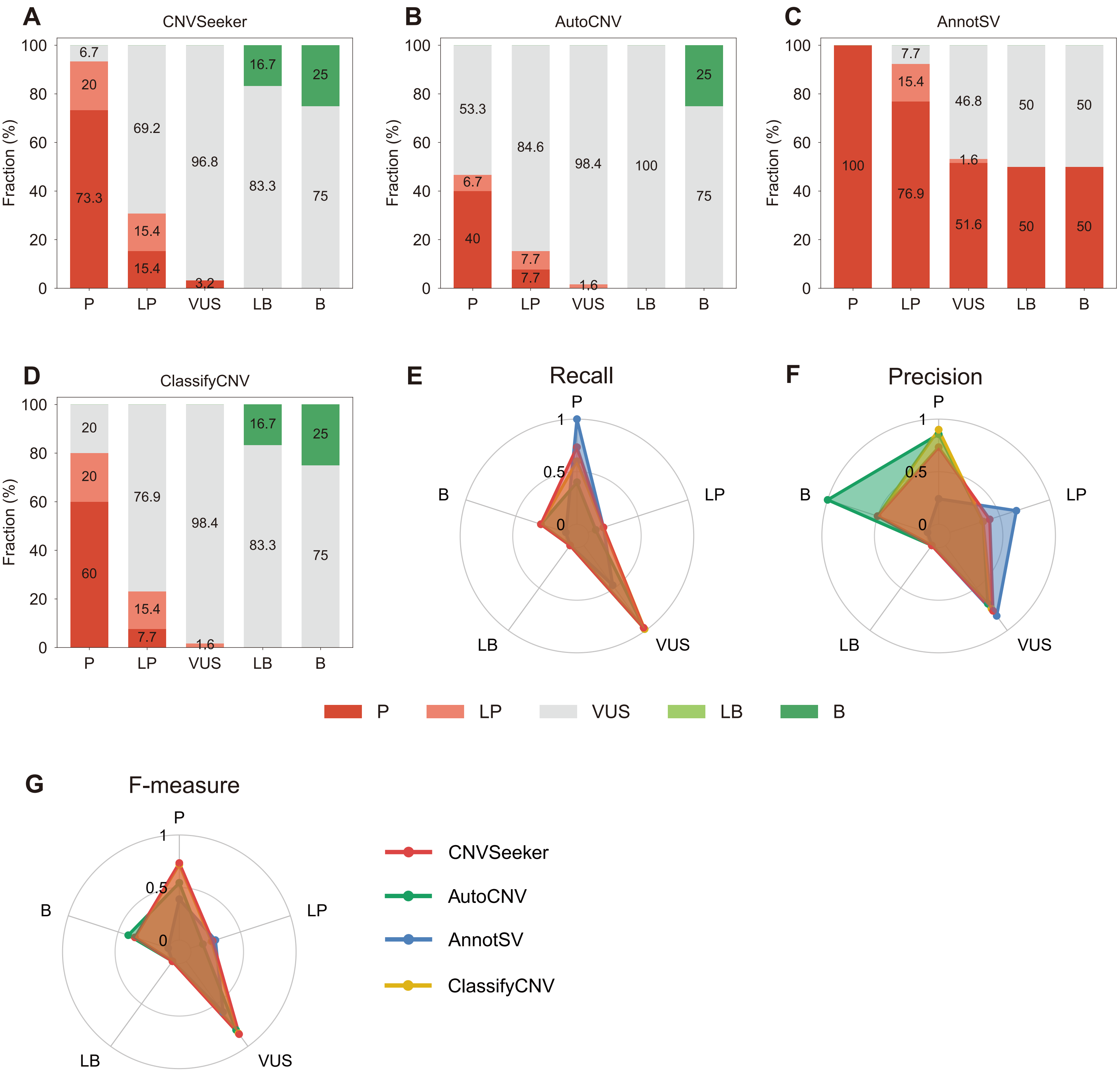

Figure S6. Performance evaluation of CNV classification by different classifiers using 100 CNVs curated from the ACMG/ClinGen original study. (**A–D**) Stacked bar plots showing the proportion of CNV classes predicted by each method, including Pathogenic (P), Likely Pathogenic (LP), Variant of Uncertain Significance (VUS), Likely Benign (LB), and Benign (B). Bars represent the fraction of CNVs assigned to each category by CNVSeeker (**A**), AutoCNV (**B**), AnnotSV (**C**), and ClassifyCNV (**D**). (**E-G**) Radar charts summarizing the recall (**E**), precision (**F**), and F-measure (**G**) for each tool across the five categories. Colors represent the four methods consistently across all panels.

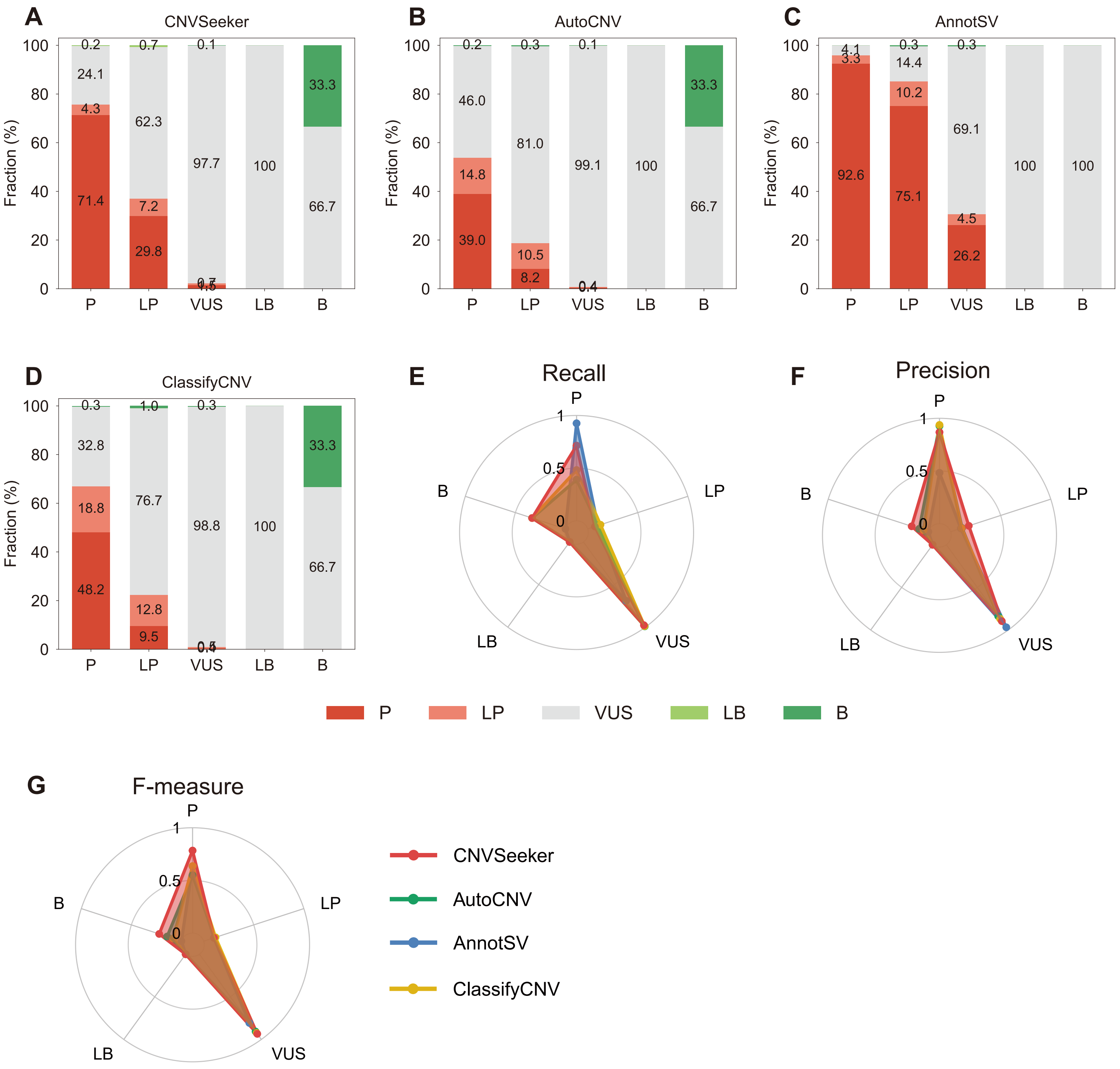

Figure S7. Performance evaluation of CNV classification by different classifiers using 6840 independent CNVs from ClinVar. (**A–D**) Stacked bar plots showing the proportion of CNV classes predicted by each method, including Pathogenic (P), Likely Pathogenic (LP), Variant of Uncertain Significance (VUS), Likely Benign (LB), and Benign (B). Bars represent the fraction of CNVs assigned to each category by CNVSeeker (**A**), AutoCNV (**B**), AnnotSV (**C**), and ClassifyCNV (**D**). (**E-G**) Radar charts summarizing the recall (**E**), precision (**F**), and F-measure (**G**) for each tool across the five categories. Colors represent the four methods consistently across all panels.

26 illustrative examples from ClinGen

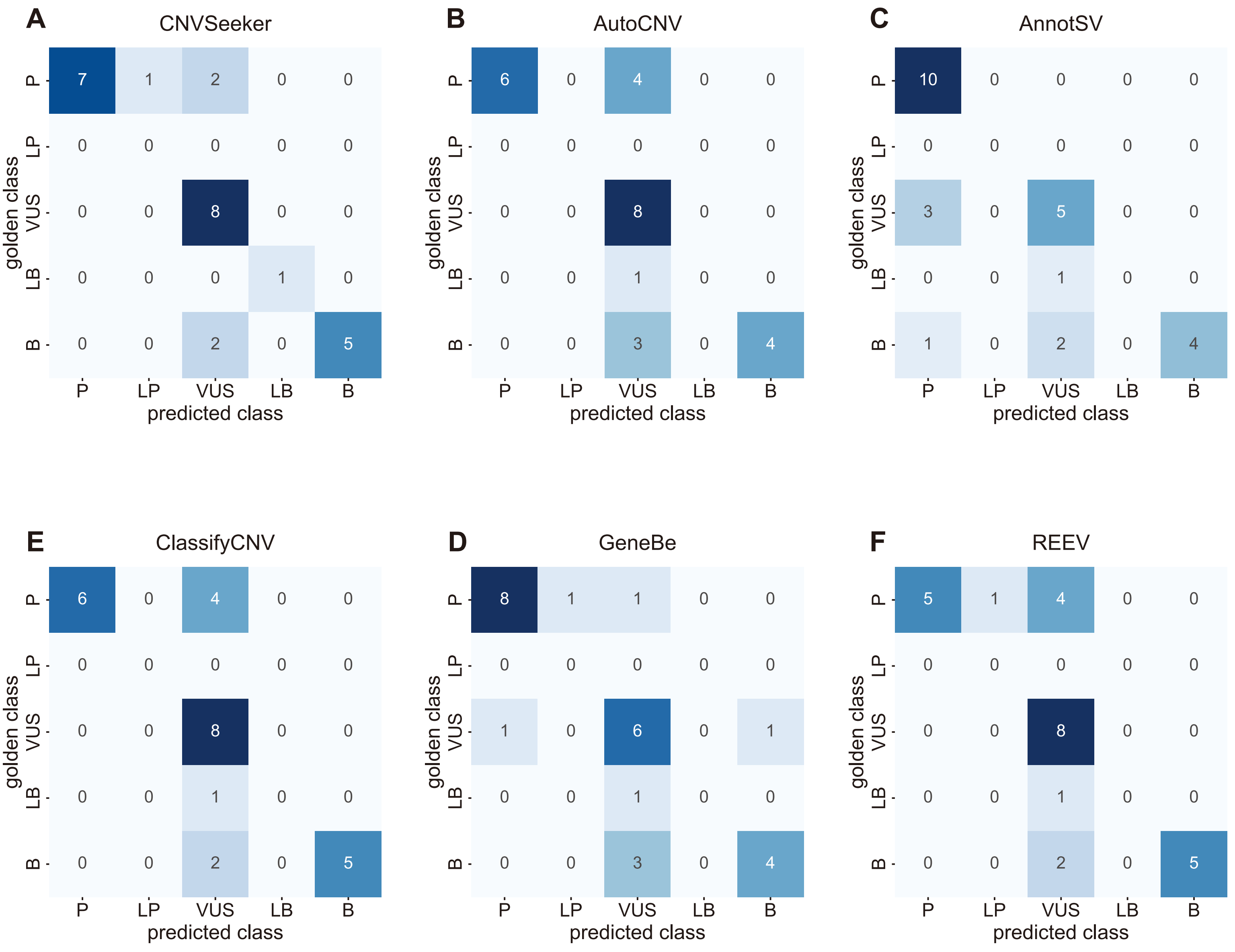

Figure S8. Confusion matrices for the performance comparison of CNV classification for different classifiers with 26 illustrative examples from ClinGen. (**A**) CNVSeeker, (**B**) AutoCNV, (**C**) AnnotSV, (**D**) ClassifyCNV, (**E**) GeneBe, and (**F**) REEV.

100 CNVs evaluated by independent reviewers from ACMG

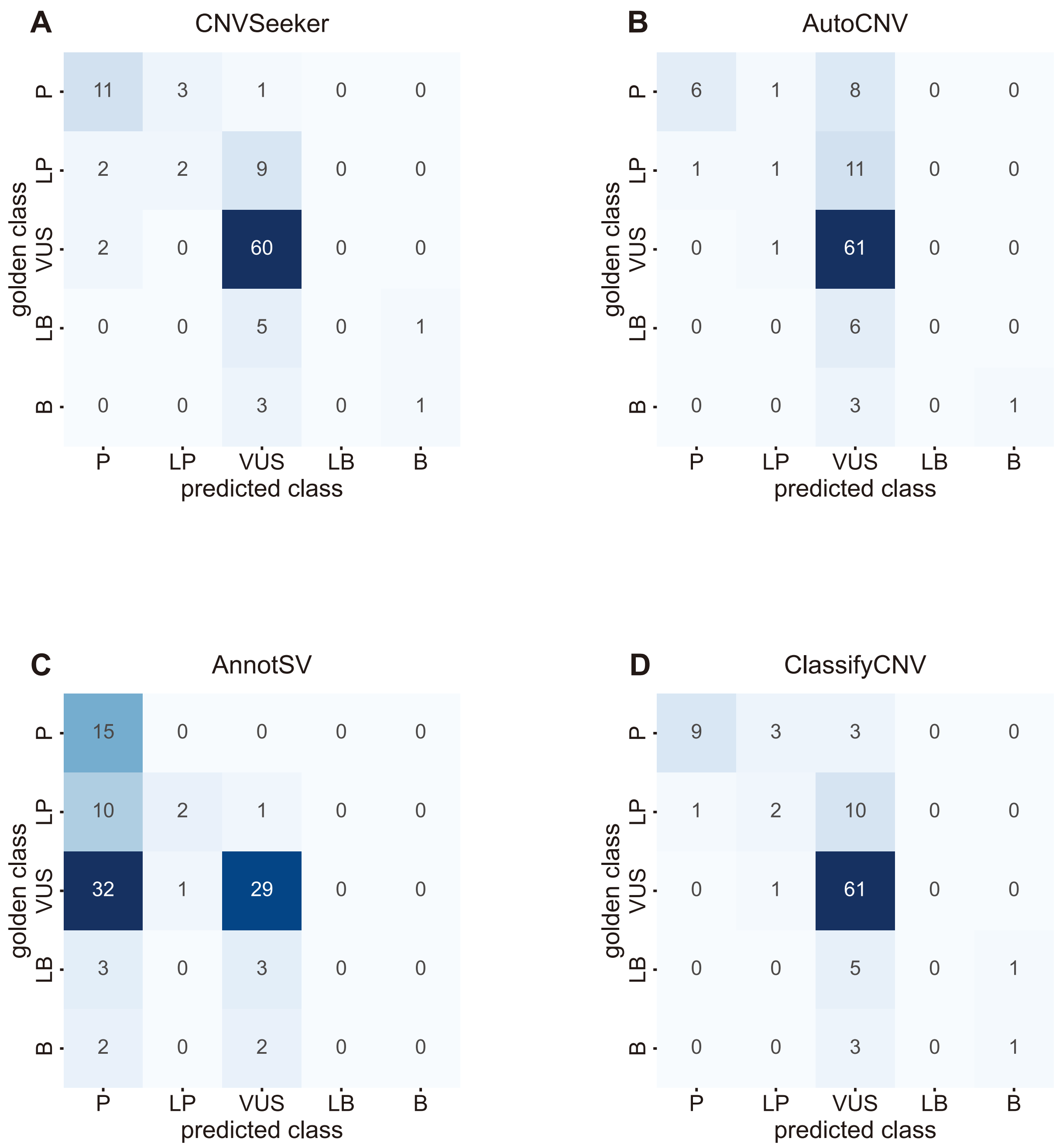

Figure S9. Confusion matrices for the performance comparison of CNV classification for different classifiers with 100 CNVs evaluated by independent reviewers from ACMG/ ClinGen originalpaper. (**A**) CNVSeeker, (**B**) AutoCNV, (**C**) AnnotSV, and (**D**) ClassifyCNV.

6840 CNVs retrieved from the ClinVar database

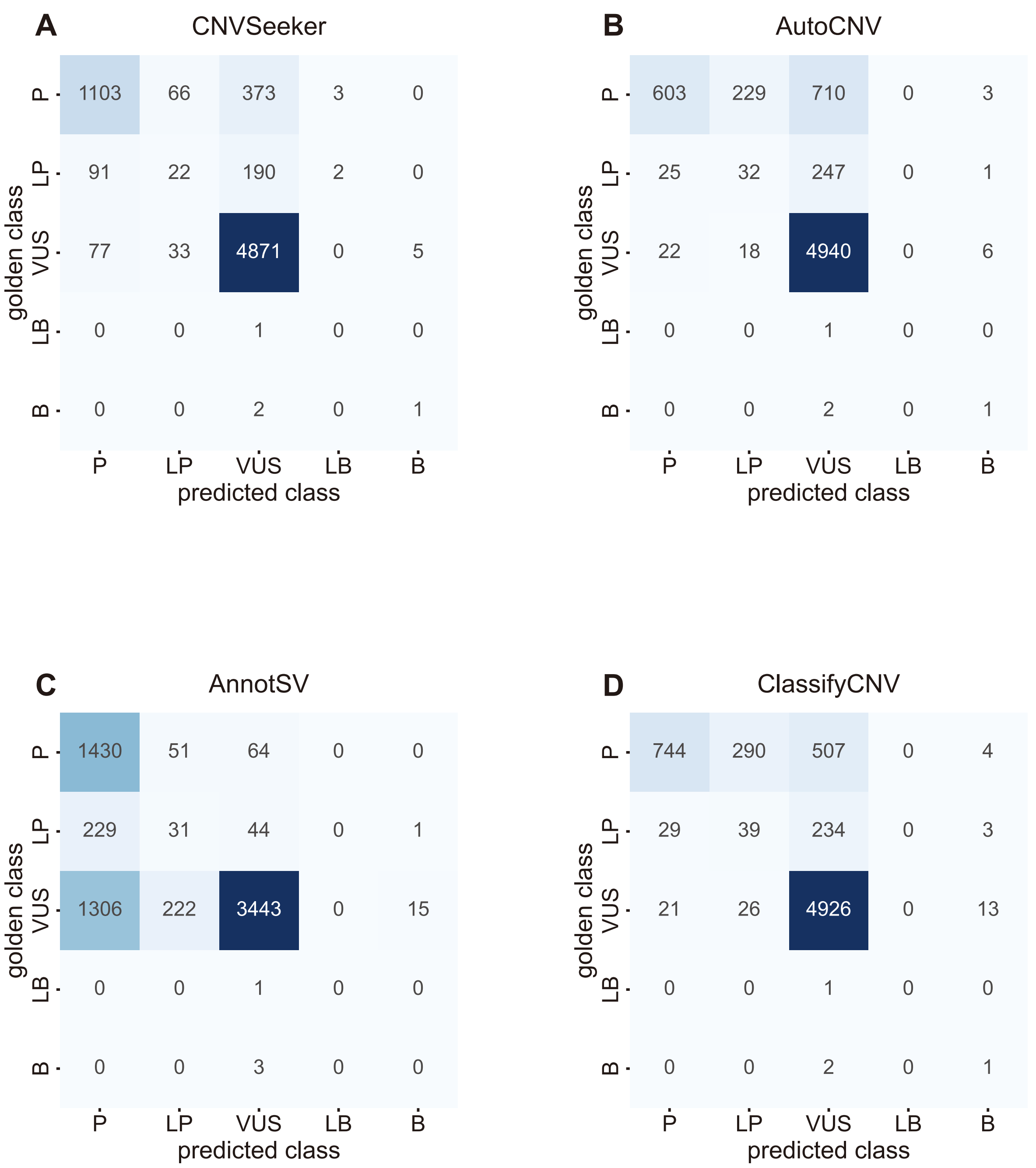

Figure S10. Confusion matrices for the performance comparison of CNV classification for different classifiers with 6840 independent CNVs cases from ClinVar. (**A**) CNVSeeker, (**B**) AutoCNV, (**C**) AnnotSV, and, (**D**) ClassifyCNV.
